# Measuring ^15^N and ^13^C Enrichment Levels in Sparsely Labeled Proteins Using High-Resolution and Tandem Mass Spectrometry

**DOI:** 10.1021/jasms.4c00237

**Published:** 2024-11-12

**Authors:** Elijah T. Roberts, Jonathan Choi, Jeremy Risher, Paul G. Kremer, Adam W. Barb, I. Jonathan Amster

**Affiliations:** 1 Department of Chemistry, University of Georgia, Athens, Georgia 30602, United States; 2 Department of Biochemistry and Molecular Biology, University of Georgia, Athens, Georgia 30602, United States; 3 Complex Carbohydrate Research Center, University of Georgia, Athens, Georgia 30602, United States

## Abstract

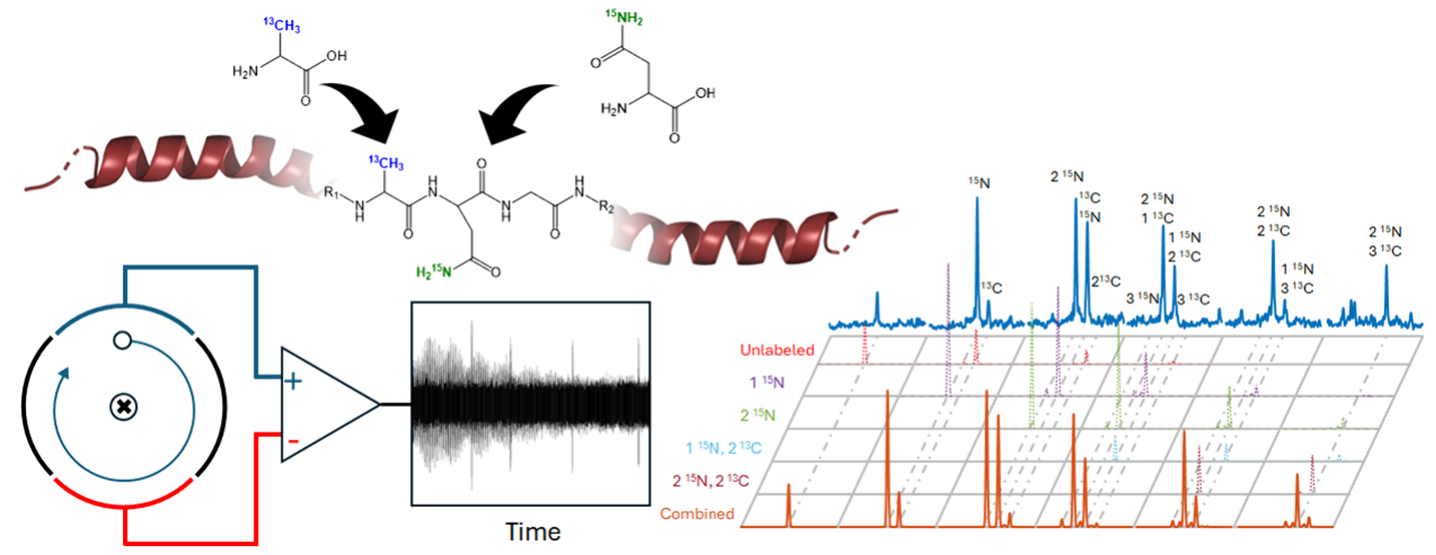

Isotope labeling of both ^15^N and ^13^C in selected amino acids in a protein, known as sparse labeling, is an alternative to uniform labeling and is particularly useful for proteins that must be expressed using mammalian cells, including glycoproteins. High levels of enrichment in the selected amino acids enable multidimensional heteronuclear NMR measurements of glycoprotein three-dimensional structure. Mass spectrometry provides a means to quantify the degree of enrichment. Mass spectrometric measurements of tryptic peptides of a selectively labeled glycoprotein expressed in HEK293 cells revealed complicated isotope patterns which consisted of many overlapping isotope patterns from intermediately labeled peptides, which complicates the determination of the label incorporation. Two challenges are uncovered by these measurements. Metabolic scrambling of amino groups can reduce the ^15^N content of enriched amino acids or increase the ^15^N in nontarget amino acids. Also, undefined, unlabeled medium components may dilute the enrichment level of labeled amino acids. The impact of this unexpected metabolic scrambling was overcome by simulating isotope patterns for all isotope-labeled peptide states and generating linear combinations to fit to the data. This method has been used to determine the percent incorporation of ^15^N and ^13^C labels and has identified several metabolic scrambling effects that were previously undetected in NMR experiments. Ultrahigh mass resolution is also utilized to obtain isotopic fine structure, from which enrichment levels of ^15^N and ^13^C can be assigned unequivocally. Finally, tandem mass spectrometry can be used to confirm the location of heavy isotope labels in the peptides.

## Introduction

Glycoproteins play a significant role in biology and medicine. These are proteins that are covalently modified cotranslationally or post-translationally with carbohydrates (glycans). Glycans themselves serve as customizable moieties, which are tailored for specific binding to receptors and other scaffolding proteins, and their presence is essential for the proper function of many proteins. Glycoproteins are found in the endoplasmic reticulum, in Golgi bodies, at the cell surface, and in the extracellular matrix, adopting important roles in cell signaling pathways, immune system function,^1,2^ cell and tissue adhesion, and early cell differentiation and cell death.^3,4^ A substantial proportion of human proteins (34%) have at least one glycosylation site, of which the majority are N-linked glycoproteins. Changes in glycosylation are implicated in diseases like cancer^5^ and inherited congenital and genetic disorders,^6^ and glycoproteins also play important roles in the mechanisms of bacterial and viral infections.^7^ Many biotherapeutic drugs are likewise proteins, including antibodies,^8^ cytokines,^9^ and blood clotting factors.^10^ As such, there is a need to study these proteins to better understand the mechanisms of diseases and develop new therapeutics.

NMR spectroscopy is an important tool for studying the structure and motion of proteins and their interactions with the environment in solution. The majority of protein expression for NMR spectroscopy is performed with *Escherichia coli*, a prokaryote that proliferates rapidly and grows on chemically defined media, allowing incorporation of ^13^C and ^15^N labels from simple and inexpensive molecules like ammonium chloride and glucose. A significant limitation of prokaryotic expression systems is that they lack the necessary cellular machinery for glycosylation, which is essential for the proper folding and function of many eukaryotic proteins.^1,11^ Glycoproteins can be expressed in mammalian cell systems, which require complex growth media. Uniform labeling of proteins in eukaryotic cell lines is considerably more costly and challenging than in prokaryotic systems.^12^ A more tractable alternative is to use sparse labeling. With this approach, cells are grown in an enriched medium with mostly unlabeled amino acids but with selected amino acids that have ^15^N- and/or ^13^C-labeled atoms in specific locations, for example, ^15^N-valine or ^13^C-methyl-alanine.

The signal level in a one-dimensional NMR experiment is directly proportional to the percentage of an NMR-active isotope at a particular site. For multidimensional experiments, the signal is proportional to the product of the labeled nuclei excited by a particular NMR experiment, and thus, high incorporation percentages are essential to provide high signal intensities. Metabolic scrambling during protein expression limits the isotope incorporation. Metabolic scrambling is a process by which the host organism metabolizes components in the growth medium, like [^15^N]-serine, to other molecules, like [^15^N]-glycine. In many situations, scrambling is undesirable because it can likewise generate [^14^N]-serine from [^14^N]-glycine in the growth medium. This situation is further complicated when unlabeled amino acids are synthesized from other unlabeled medium components, reducing signal intensity and increasing the number of signals in an NMR spectrum.^13^ However, if the locations of scrambling can be determined along with the incorporation at those sites, the information can be used to assign the extraneous peaks in the spectra as well as optimize expression conditions to minimize metabolic scrambling.

To optimize the production of sparsely labeled proteins, analytical tools are required to assess the enrichment level in the targeted amino acids. Unlike uniform labeling, the percent incorporation of selective isotope labels cannot be measured using isotope ratio MS (IR-MS). While IR-MS can provide precise and accurate enrichment levels in uniformly labeled samples, it gives a global average of the enrichment of all carbon or nitrogen^14^ For sparsely labeled proteins, we are interested in knowing the enrichment level in specific amino acids. Conventional MS measurements are more suitable for determining the percent incorporation of these labels, as they should include predictable mass shifts in peptides that contain the target amino acids. Stable isotope labeling using amino acids in cell culture (SILAC) is already employed to enhance proteomics experiments using MS.^15^ SILAC experiments typically use amino acids with more than six isotope labels, so the isotope patterns of the light and heavy peptides will not overlap, and quantitation can be performed simply by measuring the abundance of the monoisotopic peak of each species.^15^ The predictable mass shifts can also be used as tandem mass tags (TMTs) for data-dependent acquisition (DDA) experiments^16^ and as tools for quantitative proteomics.^17^

Recently Subedi et al. reported the characterization of human embryonic kidney (HEK) 293F cells with regard to their incorporation and metabolic scrambling of selectively ^15^N-labeled amino acids during protein expression for NMR.^18^ The model system used for these experiments was the highly glycosylated Fc γ receptor (FcγR)IIIa/CD16,^19^ which was expressed as a fusion protein with green fluorescent protein (GFP), connected by a tobacco etch virus (TEV) protease cleavage site. CD16 was cleaved from GFP and used for NMR experiments, while the non-glycosylated GFP was used to assess labeling behavior using MS. After some refinement, two labeling strategies were generated that demonstrated predictable metabolic scrambling, which will be referred to as VIL and KGS for the amino acids that are enriched in ^15^N and/or ^13^C. Previously, we were able to use mass spectra of labeled GFP peptides (which were not glycosylated) to determine the percent incorporation of ^15^N labels at 30 ± 14% and 52 ± 2% for the KGS and VIL constructs,^18^ but we were limited to peptides containing only one ^15^N label. Peptides which contained more than one ^15^N atom and peptides with ^13^C labels were not analyzed. Because each peptide contained only one ^15^N, the isotope patterns for the light and heavy peptides overlapped with each other; therefore, the ratio of their monoisotopic peaks could not be measured directly. To determine the percent incorporation of the label, we modified MATLAB’s isotope pattern simulator, isotopicdist, to simulate selectively isotope-labeled molecules. Then linear combinations of the simulated isotope patterns for light and heavy peptides were fit against their observed isotope patterns, and their linear coefficients were interpreted as their percent incorporation.

Here we present an approach for fitting multiple overlapping isotope pattern simulations to experimental data in order to determine the percent incorporation of multiple ^15^N and ^13^C labels using modifications to isotopicdist that allow it to simulate isotopically enriched peptides from sparsely labeled proteins. Additionally, we present the analysis of additional VIL and KGS peptides that contain more than one ^15^N label, peptides with both ^15^N and ^13^C labels, and peptides that have undergone metabolic scrambling to yield complex mixtures of enrichment levels.

## Methods

### Selectively Isotope-Labeled GFP-CD16a

All reported measurements were made on a tryptic digest of an overexpression of a fusion of green fluorescent protein (GFP) and the soluble extracellular domain of CD16a, joined by a TEV cleavage site. The cleaved GFP and CD16 were separated using size exclusion chromatography (SEC), and the GFP-containing fractions were used for MS analysis. Selectively isotope-labeled GFP-CD16a constructs were expressed as described by Subedi et al.^18^ While the two proteins were separated, some CD16a peptides were identified and analyzed, although they were not glycosylated.

Two separate constructs of GFP-CD16 were analyzed. One had labeled Val, Ile, and Leu (VIL), where each amino acid contained a ^15^N and all five carbons in Val were replaced with ^13^C. The second construct had labeled Lys, Gly, and Ser (KGS), where the Lys contained two ^15^N atoms (in the α-amino and ε-amino groups), Ser contained one ^15^N atom, and Gly contained one ^15^N and two ^13^C atoms.

GFP-CD16a (“//**//” denotes the TEV cleavage site, and glycosylation sites are marked in **bold**): MHHHHHHHHMSGLNDIFEAQKIEWHEMSKGEELFTGVVPILVELDGDVNGHKFSVRGEGEGDATNGKLTLKFICTTGKLPVPWPTLVTTLTYGVQCFSRYPDHMKRHDFFKSAMPEGYVQERTISFKDDGTYKTRAEVKFEGDTLVNRIELKGIDFKEDGNILGHKLEYNFNSHNVYITADKQKNGIKANFKIRHNVEDGSVQLADHYQQNTPIGDGPVLLPDNHYLSTQSVLSKDPNEKRDHMVLLEFVTAAGITHGEFSSENLYFQ//**//GRTEDLPKAVVFLEPQWYRVLEKDSVTLKCQGAYSPED**NST**QWFH**NES**LISSQASSYFIDAATVDDSGEYRCQT**NLS**TLSDPVQLEVHIGWLLLQAPRWVFKEEDPIHLRCHSWKNTALHKVTYLQNGKGRKYFHHNSDFYIPKATLKDSGSYFCRGLVGSK**NVS**SETV**NIT**ITQG^18^

Analysis of tryptic peptides from the unglycsolylated GFP portion of the fusion protein avoided complexities that would arise from analyzing heterogeneous N-glycosylated peptides from the Fc γ receptor. However, one could focus analysis on a glycoprotein by first treating it with PNGase F to trim heterogeneous glycsolyations to a single *N*-acetylglucosamine residue prior to tryptic digestion and MS analysis.

### MALDI-FTICR Mass Spectrometry

Mass spectra were collected on a Bruker SolariX XR 12 T Fourier transform ion cyclotron resonance (FTICR) mass spectrometer equipped with a dual ESI/MALDI ion source and a dynamically harmonized ParaCell. The MALDI source was equipped with a SmartBeam II laser. The laser power was set to 65%, and between 5 and 50 laser shots were used to adjust the total ion count (TIC). Spectra were collected between *m*/*z* 500 and 3000 with 512k data points and a 0.6991 s transient, which gave a resolution of 80,000 at *m*/*z* 1000; 48 scans were collected per spectrum. Mass calibration for protein digests was performed with cesium iodide (CsI) (Aldrich 99.9999%) between *m*/*z* 500 and 3000 using ESI with an RMS error of ∼0.15 ppm.

### Capillary Zone Electrophoresis–Mass Spectrometry (CZE-MS)

CZE-MS experiments were performed using an ECE 001 capillary electrophoresis instrument (CMP Scientific, Brooklyn, NY) coupled to a Bruker SolariX XR 12 T FTICR instrument using an EMASS-II sheath flow CE-MS interface (CMP Scientific). CZE separations were performed on a 55 cm bare-fused silica (BFS) capillary. The background electrolyte was 5% v/v formic acid (reagent source and grade) in HPLC-grade H_2_O. Sample injection was performed with 200 mbar pressure for 10–30 s depending on the run. This gave injection volumes between 60 and 120 nL, which occupied 6–17% of the total capillary volume. Samples were dissolved in 25 mM ammonium bicarbonate (reagent source and grade) to perform pH stacking prior to the CZE separation.^20^ A voltage of 30 kV was applied to the capillary for 30 min to achieve separation. The sheath liquid (SL) in the CZE-MS interface was 90:10:0.1 v/v/v H_2_O/methanol/formic acid, and a voltage of 2 kV was applied to the sheath liquid reservoir to drive electrospray.

For CZE-MS experiments where isotopic fine structure was not resolved, including online MS/MS experiments, mass spectra were collected on the 12 T FTICR between *m*/*z* 150 and 3000 with a transient length of 0.8389 s, which gave a resolution of 90,000 at *m*/*z* 1000. Accumulation was set to 0.05 or 0.1 s. MS/MS was performed using CID with a top 2 data-dependent acquisition. CID was performed in the hexapole ion trap/collision cell, and the collision voltage was set to either 15 or 20 V. MS1 accumulation was set to 0.05 s, and MS2 accumulation was set to 0.7 s. A preferred *m*/*z* list was also generated that contained the masses of all of the fully labeled peptides, and the quadrupole isolation window was set at *m*/*z* 15 to ensure that the peptides in all states between unlabeled and fully labeled were transmitted simultaneously.

Isotopic fine structure was obtained during CZE-MS experiments by performing parallel absorption mode processing on an FTMS booster (Spectroswiss, Lausanne, Switzerland). Identical transient lengths were used as described above, but absorption mode processing gave an additional doubling in mass resolution over standard magnitude mode processing.

### Isotopic Fine Structure (nESI)

To obtain isotopic fine structure in direct infusion experiments, mass spectra were collected in broadband mode between *m*/*z* 600 and 3000 with 8M points and a 13.4218 s transient. Labeled peptides were individually isolated with the quadrupole to minimize space charge effects and maximize signal-to-noise (SN), and resolving powers between 800k and 2M were observed depending on the MW and charge of the peptide. Twenty-four scans were averaged per spectrum. Samples were introduced using nanoelectrospray ionization (nESI). Borosilicate nESI emitters were pulled in house with a Sutter p1000 micropipette puller. Fire-polished, filamented capillaries (OD 1.2 mm, ID 0.69 mm, 10 cm; item BF120-69-10) were pulled to an orifice size of ∼1 μm. Approximately 10 μL of sample was loaded into the emitter, which was grounded with a platinum wire. A voltage of 1100–2000 V was applied to the inlet capillary of the MS to achieve electrospray. Absorption mode processing was also performed in parallel using the FTMS booster (Spectroswiss), which gave resolutions between 1.6M and 4M.

### Simulating Sparsely Labeled Peptides

Simulations of selectively isotope-labeled peptides were generated with a modified version of MATLAB’s isotope simulator, isotopicdist, which is based on Rockwood’s Fourier transform (FT) algorithm for simulating isotope patterns.^21^ Two pairwise inputs, “Csparse” and “Nsparse”, were added to isotopicdist to allow for the addition of selective ^13^C or ^15^N labels into the elemental formula prior to simulation. In our previous paper, we described a method to simulate uniform isotope labeling by altering the abundances of various isotopes; however, selective isotope labeling requires modifying the elemental formula (M), the table of isotope masses for each element (A), and the table of isotopic abundances (B). The default values for these three variables are shown below for a simulated peptide “PEPTIDE” with elemental formula C_35_H_53_N_7_O_15_.
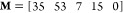

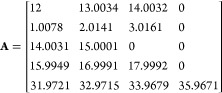

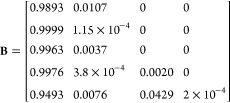
where **M** is the elemental formula vector, with the values presented in the order C H N O S, **A** is the table of isotope masses for CHNOS, and **B** is the table of isotopic abundances for CHNOS. To add a ^13^C label, a new column is added to the elemental formula vector with a 1 to represent the ^13^C label, effectively treating it as its own element, and 1 is subtracted from the initial carbon count. New rows are likewise added to the **A** and **B** matrices to contain the mass and abundance of the “new element”, which in this case are 13.00335 Da with 100% abundance:
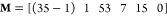

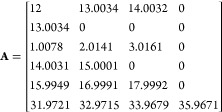

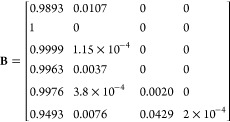
The process for adding a selective ^15^N label is identical, only the count for N is modified and the mass of the label is the mass of ^15^N. ^13^C and ^15^N labels can also be added simultaneously. Some example calls of the isotopicdist function to add a single ^13^C label, a single ^15^N label, and one of each label are “isotopicdist(f, ‘Csparse’, 1)”, “isotopicdist(f, ‘Nsparse’, 1)”, and “isotopicdist(f, ‘Csparse’, 1, ‘Nsparse’, 1)”, respectively, where f is the elemental formula to be simulated.

### Basic Peptide Mass Fingerprint Search

In order to quickly profile digests of selectively isotope-labeled proteins, a rudimentary peptide mass fingerprinting script was developed in MATLAB that can accept custom amino acid labeling parameters. The script accepts a simple masslist file (.asc), a list of tryptic peptides to assess, a list of amino acids that contain labels, and a matrix that contains the number of ^13^C and ^15^N labels in each amino acid. Finally, the script also accepts a full profile (.xy) spectrum as an input to generate figures with overlays of the simulated and observed isotope patterns following the mass fingerprint search.

First, the program counts the number of amino acids that should contain labels, and then the numbers of ^13^C and ^15^N labels in each peptide are counted based on the user-specified labeling properties. Next, isotope patterns are generated for the unlabeled and fully labeled peptides. Finally, the monoisotopic masses are extracted from each of the simulations and used to search the data. In practice, this is not much different than adding sparse isotope label standard proteomics database searches with Protein Prospector, Mascot, or Byonic. We have found that the options for isotopically labeled amino acids are limited in these programs, but our program allows the user to quickly change the number and type of labels in each amino acid. This also allows users to generate labeled amino acids that are not commercially available and which might only arise from metabolic scrambling in the expression system.

If a match is found within the user-specified error tolerance, then it is reported in an output table, and the script automatically generates a figure that overlays the unlabeled and labeled isotope pattern simulations with the observed isotope pattern in the MS data. This allows for visual inspection of the observed isotope patterns, which is particularly useful when the incorporation of the label is incomplete or if there are metabolic scrambling effects at play.

### MS/MS Sequencing of Labels

To sequence the locations of each label using MS/MS data, a similar process as described above is used. Rather than assessing a list of tryptic peptides, the user-defined labeling rules can be used to assess a list of B and Y fragments for a defined peptide (Figure S1). First, the user inputs MS data in .asc format before, in addition to the peptide sequence, charge, mass error tolerance, and amino acid labeling rules. The script calculates all C-terminal and N-terminal sequences for the given peptide and then calculates all of the possible B and Y ion formulas for each sequence. Lastly, all possible monoisotopic masses for each B and Y ion for all charges up to that of the precursor are calculated, and this list is used to search for matching fragment ions in the MS data. As before, figures are procedurally generated that display the isotope patterns and simulations for each match so that they may be visually inspected. While this process does not currently support the analysis of fragment ions generated from ETD, ECD, or UVPD, it could reasonably be upgraded to do so. Supplementary peptide sequencing was also performed with Prosight Lite and Protein Prospector to confirm the identities of unlabeled fragment ions.

### Determining Percent Incorporation for Multiple Labels

Following either the peptide mass fingerprint search or the peptide fragment analysis described above, the percent incorporation of the labels can be determined using isotope pattern simulations. To determine the percent incorporation of the isotope label, the ratio of the labeled peptide to the unlabeled peptide must be determined. However, there are many circumstances where the isotope pattern of the labeled peptide overlaps with isotope peaks from the unlabeled peptide, so a simple ratio of the monoisotopic peaks from each species will not provide an accurate result. Instead, we generate linear combinations of the labeled and unlabeled peptides’ isotope patterns and fit them to the data using root-mean-square error (RMSE). The linear combination isotope pattern *F*(*m*)_c_ is represented by
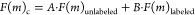
1where *A* and *B* are linear weighting coefficients such that *A* + *B* = 1, *F*(*m*)_unlabeled_ is the isotope pattern of the unlabeled peptide in full profile mode, and *F*(*m*)_labeled_ is the isotope pattern of the labeled peptide in full profile mode. *F*(*m*)_c_ are iteratively generated and fit to the MS data using RMSE, ranging from 0% labeled to 100% labeled, and the *B* coefficient for the best fitting *F*(*m*)_c_ is taken as the percent incorporation for that label. This approach works only if there is a single labeled amino acid, for example, if a ^15^N label was added to the Ile in “PEPT**I**DE”. If there is more than one label present, or there are unintended intermediates due to metabolic scrambling, more terms need to be added to calculate *F*(*m*)_c_:
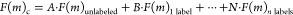
2where *F*(*m*)_1 label_ is a simulated isotope pattern for a peptide containing only one additional label and *F*(*m*)_*n* labels_ is a simulated isotope pattern for the peptide containing the full number (*n*) of intended isotope labels. As an example, if a labeled peptide contains four separate ^15^N labels, simulations would be generated for the unlabeled peptide as well as the peptides containing one, two, three, or four labels.

If both ^13^C and ^15^N labels are present, calculating all possible intermediate states is more complicated. The user can define the interval at which each ^15^N and ^13^C label can be removed to create an intermediate state. For example, if there are labeled Val with one ^15^N and five ^13^C, the user would define this interval as [1,5] because the ^15^N could be removed via metabolic scrambling and, if the ^13^C were omitted, it is likely that they would all be included or none of them would. So, for the peptide “AAAVVVAAA”, which contains three labeled Val, 12 intermediately labeled distributions would be generated in addition to the unlabeled and fully labeled distributions. Before the fitting process starts, the user is prompted to review these labeling possibilities and may add or remove intermediate states or even add extra labels beyond the initial value.

To calculate the weighting coefficients in eq 2, many *F*(*m*)_c_ are iteratively generated and fit to the data (Figure S2). First, the script determines the lowest-MW species that is present, which is usually the unlabeled distribution, and scales the simulated distribution to its nearest-neighbor peak in the spectrum by adjusting *A* in eq 2. Then the scaling coefficient for the next isotope pattern is iteratively increased until the monoisotopic peak of the *n*th isotope pattern matches the intensity of its nearest neighbor in the spectrum. This process is repeated for each isotope pattern until all of the patterns have been assessed and scaled. Finally, once all weighting coefficients have been determined, they are normalized so that their sum equals 100. The normalized coefficients can then be taken as percent abundances for each state of labeling. It should be noted that this process only works automatically for peptides where no two intermediates add the same number of heavy isotope labels, which is prone to happen for larger peptides with both ^15^N and ^13^C labels. If that is not the case, then the intermediate states must be manually reviewed, which is described below.

The same fitting process described above can easily be performed using simulations of the isotopic fine structure, which helps greatly to discriminate between ^15^N and ^13^C labels. Isotopicdist can generate simulated distributions at any user-defined resolution, all the way down to isotopic fine structure. The script measures the fwhm peak width for the peptide currently being fit and then generates the simulated isotope patterns with the same peak width before the fitting process begins, so no additional user input is required to fit fine-structure data. In addition, higher point density is required for the FFT (10k points/Da) to simulate peaks with Gaussian profiles, and the standard setting of 1000 points/Da will generate triangular peak shapes for fine-structure simulations.

## Results and Discussion

In prior work with Subedi et al., the percent incorporation of ^15^N labels in expressions utilizing labeled Val, Ile, and Leu (VIL) or Lys, Gly, and Ser (KGS) were determined for peptides that contained only one label using the method described in eq 1.^18^ From tryptic digests of the VIL and KGS constructs, peptides that contained a single ^15^N-labeled amino acid were selected and fit using eq 1 to determine the percent incorporation of the label. We were able to provide an estimate for the incorporation of ^15^N labels of 30 ± 14% for ^15^N-VIL and 52 ± 4% for ^15^N-KGS. In the KGS expression, the labeled Lys contained ^15^N-α and ^15^N-ε nitrogen, but Lys-containing peptides did not have the expected 2 Da shift; instead, each had a large A + 1 peak. The ε-amino group has a known pathway for exchange with the (unlabeled) amino group of glutamate, and the data supported that such exchange occurs quantitatively. Analysis assuming a single labeled nitrogen in the lysine produced a good fit of the data, with an enrichment level of 52%.^18^

**Figure 1:**
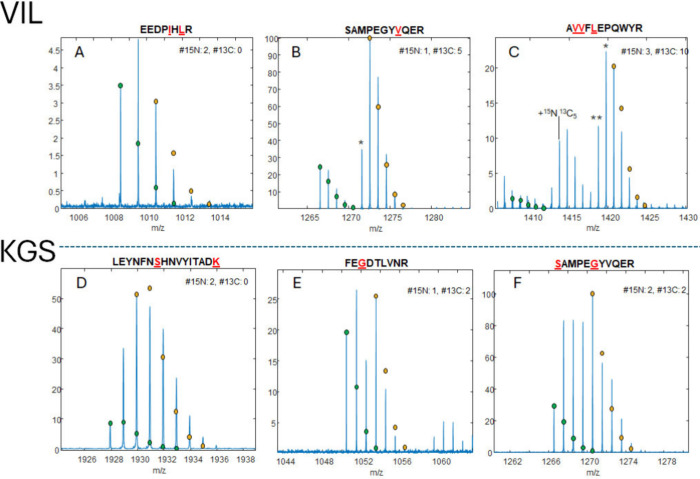
Mass spectra (blue) of selectively isotope-labeled peptides from (A–C) the VIL construct and (D–F) the KGS construct. The VIL construct contains ^15^N-Ile, ^15^N-Leu, and [^15^N, ^13^C_5_]-Val, and the KGS construct contains ^15^N-Lys, [^15^N, ^13^C_2_]-Gly, and ^15^N-Ser. Simulations for the unlabeled peptides are colored green, and simulations for the labeled peptides are shown in yellow.

Figure 1A–C and Figure 1D–F respectively show selected tryptic peptides from the VIL and KGS sparse labeling of the GFP-CD16 fusion protein that contain more than one ^15^N label or a combination of ^15^N and ^13^C labels. Complete results for the VIL peptides are available in Figures S3 and S4 and Table S1, and complete results for the KGS peptides are available in Figure S5 and S6 and Table S2. From these mass spectra, we can infer that in all cases the incorporation of labels was incomplete, and there are many overlapping isotope patterns from unlabeled and incompletely labeled peptides. Simulations for the unlabeled peptides (green) and fully labeled peptides (yellow) are overlaid on the data and scaled to their respective monoisotopic peaks. As can be seen in Figure 1, simply scaling the labeled simulation to its corresponding peak in the data produces a suboptimal fit because the abundances of many peaks are the result of overlapping contributions from isotopomers with fewer labels. The peptide EEDPIHLR (Figure 1A) should contain two ^15^N labels, but a large A + 1 isotope peak indicates that there is a significant contribution from a component with only one heavy label. Therefore, the monoisotopic peak for the fully labeled peptide also contains the A + 1 peak from the peptide with one ^15^N and the A + 2 peak from the unlabeled peptide. The contributions of each of these peaks must be considered in order to derive an accurate assessment of the percent abundance of the fully labeled peptide.

The shapes of the observed isotope patterns give qualitative insights into possible metabolic scrambling processes. The peptide SAMPEGYVQER from the VIL construct (Figure 1B) should contain one ^15^N and five ^13^C atoms if the labeled valine is incorporated as supplied in the growth medium, with an isotope distribution 6 Da higher than that of the peptide with naturally occurring isotope abundances. However, the intense peak at +5 Da (marked with an asterisk) above the monoisotopic peak suggests that a fraction of the α-amino group ^15^N on Val is lost through metabolic scrambling. The peptide AVVFLEPQWYR (Figure 1C) exhibits similar behavior. It has two [^15^N, ^13^C_5_]-Val and one ^15^N-Leu, so there are more combinations of labeled and unlabeled amino acids to consider. From the fully labeled peak, the peaks marked with “*” and “**” correspond to the loss of one and two ^15^N labels, respectively, which could result from loss of the α ^15^N from any of the labeled amino acids or the inclusion of an unlabeled Leu. Also, there are two peaks at +6 and +7 Da above the monoisotopic peak, which correspond to the inclusion of a single [^15^N, ^13^C_5_]-Val plus the addition of another ^15^N-Leu.

Similar patterns can be observed in the peptides from the KGS construct (Figure 1D–F). In most cases, the unlabeled monoisotopic peak and the fully labeled peak are visible, as well as many peaks in between, which correspond to intermediate levels of labeling. An interesting caveat here is that Ser and Gly can metabolically interconvert. The side chain of ^15^N-Ser can be removed with serine hydroxymethyltransferase (B3LMP8) to make a [^15^N]-Gly, and [^15^N, ^13^C_2_]-Gly can be converted to [^15^N ^13^C_2_]-Ser with the addition of a side chain. Both ^15^N-Ser and [^15^N, ^13^C_2_]-Ser are possible, and [^15^N, ^13^C_2_]-Gly and ^15^N-Gly might also be present. For example, the peptide FEGDTLVNR (Figure 1E) has an expected A + 3 peak but also a large A + 1 peak, which is evidence that both [^15^N, ^13^C_2_]-Gly and ^15^N-Gly contribute to the labeling. Figure 1F shows the peptide SAMPEGYVQER from the KGS construct, which has both Ser and Gly and can have all four of the aforementioned labeling possibilities in addition to possible scrambling into Ala, leading to a much more complex isotope pattern than expected. In order to determine the labeling percentages, the relative contribution of each potential labeling state must be considered, including the incorporation of unlabeled amino acids, labels lost due to scrambling, and the addition of labels into off-target amino acids due to scrambling.

### Fitting Isotope Patterns with Multiple Overlapping States

**Figure 2:**
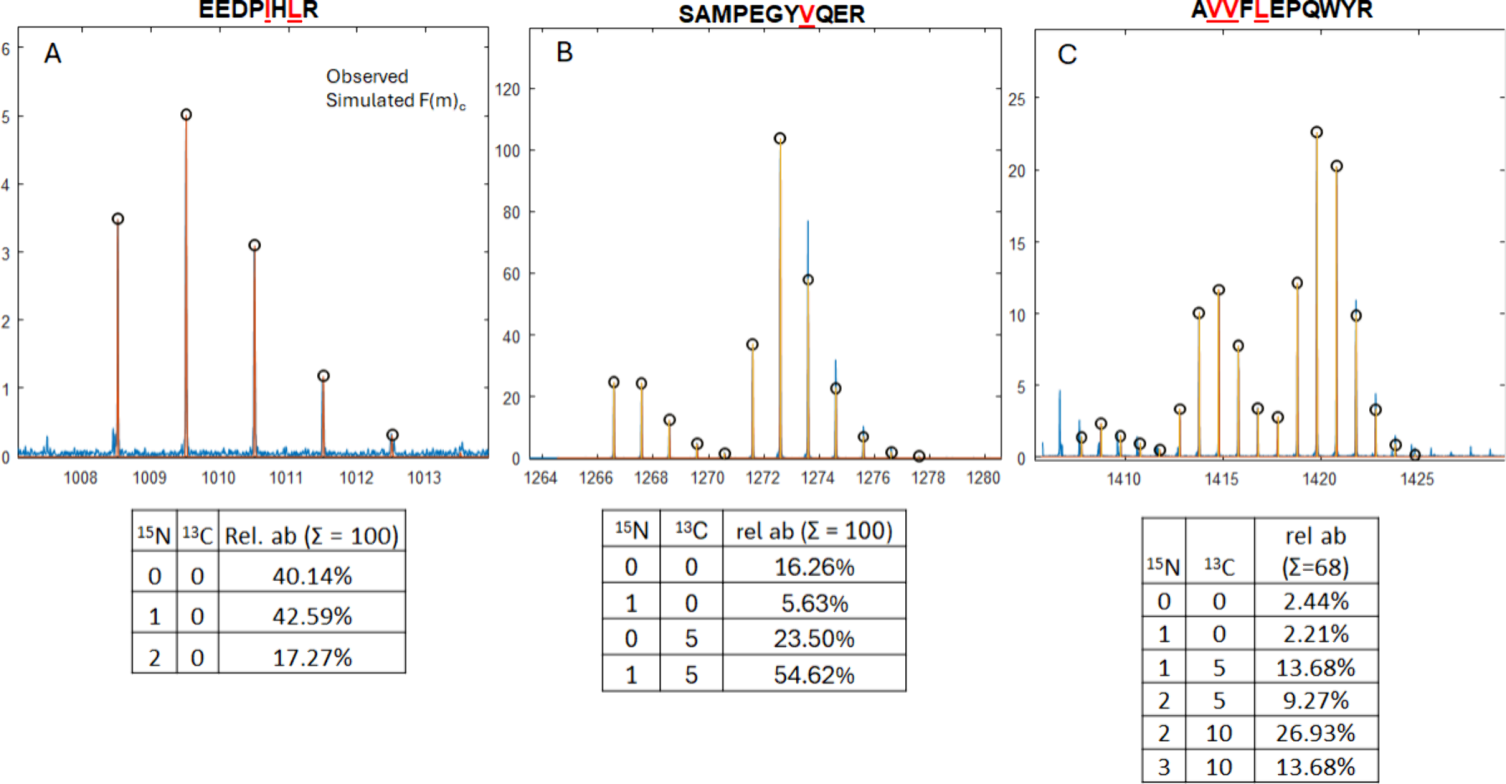
Mass spectra (blue) for selected labeled peptides from the VIL construct overlaid with a simulated isotope pattern (orange) constructed from linear combinations of several simulated labeled isotope patterns: (A) data for EEDPIHLR; (B) data for SAMPEGYVQER; (C) data for AVVFLEPQWYR. The amino acids targeted for sparse labeling are underlined and shown in red. The labeling states used to generate the combined distributions and their relative weights are shown in the tables below the corresponding spectra.

As can be seen from the data presented above, there are a few challenges in assessing the level of incorporation of isotope labels in specific amino acids. Metabolic scrambling is problematic for amino groups that can lose or gain labeled nitrogen by exchange with unlabeled or labeled amino acids, respectively. Also, some unlabeled variants of the target amino acids may be present in the growth medium and may be incorporated in competition with the labeled forms. For each of the previously shown peptides, the analysis procedure described in Methods and illustrated in Figure S2 was applied to determine the percent incorporation for each possible labeling state. This allows for the incorporations of individual labels to be determined as well as the incorporations of labeled amino acids that only appeared in peptides with more than one label. For the VIL construct, we assumed that any integer amount of ^15^N labels between unlabeled and fully labeled was possible and that for the ^13^C-labeled valine an increment of 5 was possible. This does not account for the possibility that labels can be lost due to side-chain removal on Val, but it does allow for Val to be incorporated without a ^15^N label due to metabolic scrambling. Figure 2A shows the fit for peptide EEDPIHLR (VIL). Three simulations were combined for this peptide: one unlabeled, another with one ^15^N, and a third with two ^15^N. From these calculations, 42% of the population of this peptide contains either a ^15^N-Leu or ^15^N-Ile, and 17% of the population contains both ^15^N labels, while 40% remain unlabeled.

**Figure 3:**
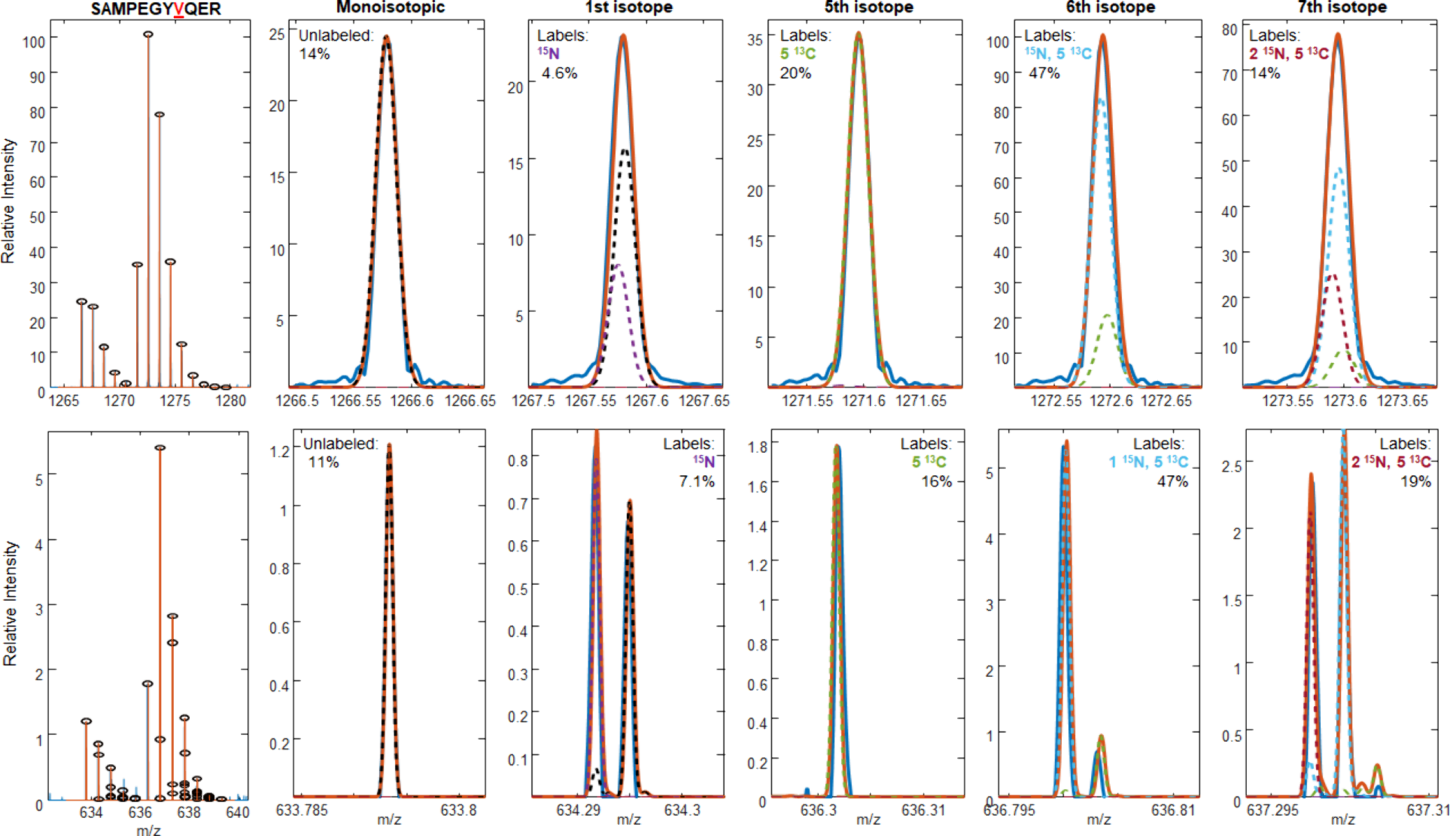
Mass spectra of the peptide SAMPEGYVQER (VIL) with unresolved fine structure (top) and resolved fine structure (bottom). The fitting process was performed with the assumption that an extra ^15^N label (dotted maroon) was added to Ala. Isotope pattern simulations for individual labeling states are shown as dotted lines, and the linear combination of all intermediate labeling states is shown in solid orange.

Figure 2B shows the results for SAMPEGYVQER, which should contain a [^15^N, ^13^C_5_]-Val; however, this is only 55% of isotopic distribution. Interestingly, almost half of the Val lost their α-amino ^15^N, shown by the peak at A + 5 in Figure 2B. 16% of the peptide was unlabeled, and a small amount of the peptide (5%) contained only a single ^15^N at an unknown location, although we hypothesize that it could be alanine, as it was previously identified as a recipient of metabolically scrambled ^15^N.^18^ The labeling possibilities do not fully account for all of the peak intensity in the A + 7 peak of the observed distribution. This could also be due to an extra ^15^N label being incorporated into Ala. If an extra ^15^N label is considered (Figure 3 top), the isotopic enrichment distribution is computed to be the following: unlabeled, 14%; ^15^N, 4.6%; ^13^C_5_, 20%; [^15^N, ^13^C_5_], 47%; and [^15^N_2_, ^13^C_5_], 14%. To confirm the contributions of the various isotopomers to the overall isotope distribution of SAMPEGYVQER, isotopic fine structure data were obtained (Figure 3 bottom). These data confirmed that the extra A + 1 contribution was from ^15^N, the A + 5 peak was due to the addition of five ^13^C, the A + 6 peak had one ^15^N and five ^13^C coming from a fully labeled Val, and the extra seventh label was a ^15^N. The estimates for the incorporation were similar to the lower-resolution spectrum and are as follows: unlabeled, 11%; ^15^N, 7%; ^13^C_5_, 16%; [^15^N, ^13^C_5_], 47%; and [^15^N_2_, ^13^C_5_], 19%.

Figure 2C shows the fitting results for the peptide AVVFLEPQWYR, which should contain two [^15^N, ^13^C_5_]-Val and one ^15^N-Leu and is one of the most complicated isotope distributions that can be fit with this process while still extracting meaningful incorporation values. In total, there are 10 intermediate labeling possibilities in addition to unlabeled and fully labeled peptides. Figure 2C only shows the labeling possibilities that are due to inclusion/omission of entire amino acids (∑ = 68%) but does not show the other possibilities that arise from removal of the α-amino ^15^N on either Val, or addition of the ^15^N into Ala (∑ = 34%). The ^15^N-Leu and unlabeled peptides contributed almost equally (∼2%) to the observed isotope pattern, corroborating the ^15^N-Leu incorporation determined in Figure 2A. The incorporation of [^15^N, ^13^C_5_]-Val was 13.68%, or 5.6 times the abundance of the unlabeled peptide, which agrees less with the value determined in Figure 2B, where the abundance of the labeled peptide was 3.4 times the abundance of the unlabeled one. The increase in abundance could be due to the presence of multiple species that contain one ^15^N and five ^13^C, like a ^13^C_5_-Val and a ^15^N-Leu or ^15^N-Ala, which would all have the same *m*/*z* and are indistinguishable without MS/MS.

For larger peptides with more labels, especially those with both ^15^N and ^13^C labels (or any heterolabels), this strategy begins to break down. For example, the peptide GEELFTGVVPILVELDGDVNGHK (Figure S9) can contain up to four labeled Val, three labeled Leu, and one labeled Ile. Accounting for all possibilities for amino acid incorporation and scrambling, there are 45 possible labeling states, which range from 0 to 28 labels (Table S4). Because there are ^15^N and ^13^C labels, 36 of these states have the same number of added neutrons as at least one other (Table S4). Currently, the software simply scans the list and assigns the first occurrence to the table. If there exist two peptides with *N* = 8 labels, either from eight ^15^N or three ^15^N and five ^13^C, whichever is in line first will be analyzed, and the second will be ignored. It is possible to determine which is the better option using the isotopic mass defect, as the peptide with more ^15^N labels shifts each isotope to lower mass, but this must be performed manually, as described below. Alternatively, if the isotopic fine structure can be obtained, then the contributions of ^15^N and ^13^C labels can be unequivocally assigned by their accurate mass.

### Discriminating between ^15^N and ^13^C Labels: Mass Defect versus Isotopic Fine Structure

**Figure 4:**
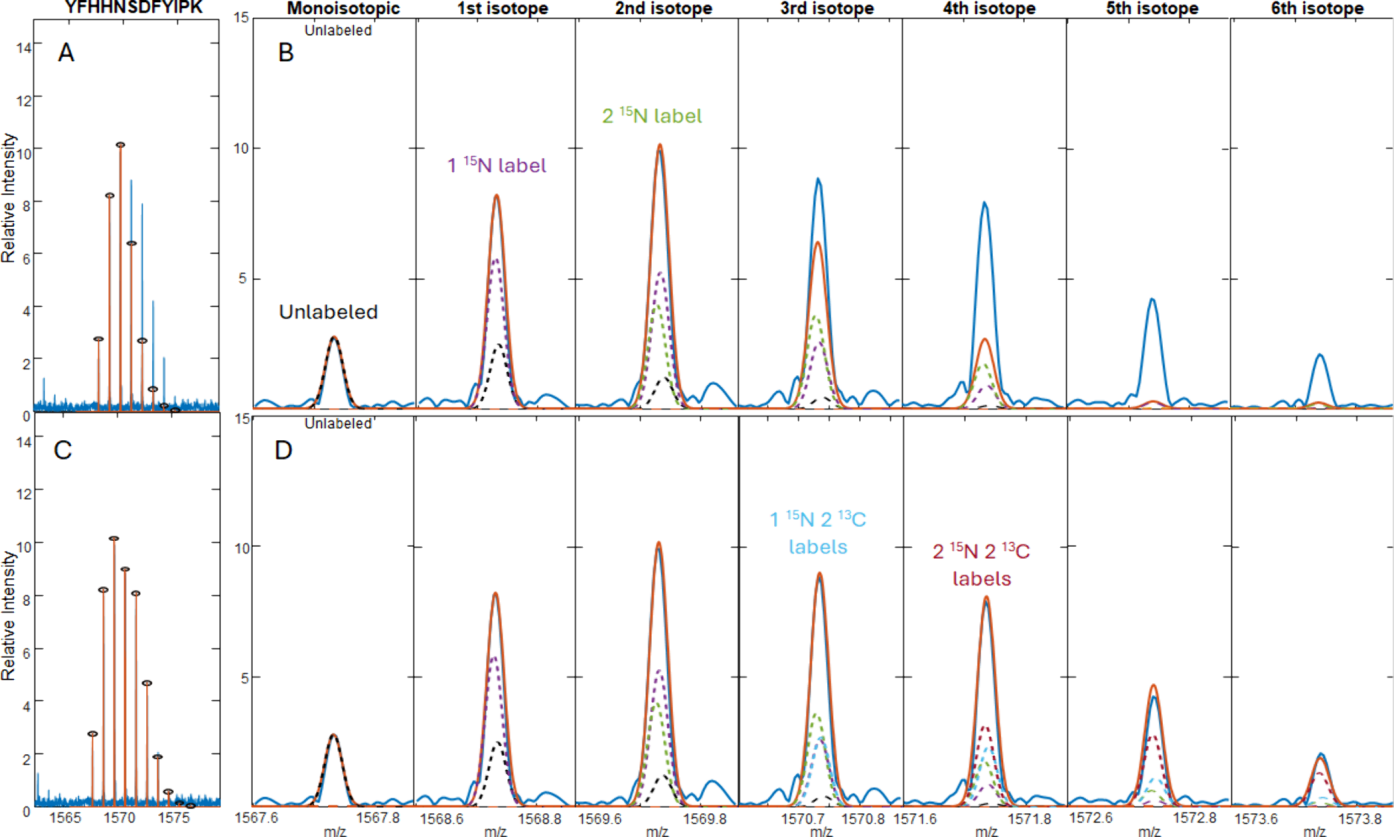
(A) Mass spectrum (blue) for the peptide YFHHNSDFYIPK overlaid with the simulated linear combination isotope pattern (orange) assuming a maximum of two ^15^N labels. (B) Expansions of each isotope peak from (A), overlaid with isotope pattern simulations for each intermediate labeling state, shown as dotted lines. (C) Mass spectrum (blue) for the peptide YFHHNSDFYIPK overlaid with the simulated linear combination isotope pattern (orange) permitting [^15^N, ^13^C_2_] and [^15^N_2_, ^13^C_2_] as additional labeling possibilities. (D) Expansions of each isotope pattern from (C), overlaid with isotope pattern simulations for each intermediate labeling state, with the addition of [^15^N, ^13^C_2_] (dotted cyan) and [^15^N_2_, ^13^C_2_] (dotted maroon) labels.

As previously discussed, for any given peptide, we observed a fully labeled and an unlabeled version as well as several intermediately labeled states. However, for peptides with both ^15^N and ^13^C, particularly larger peptides, there are intermediate states where there are multiple ^15^N and ^13^C combinations that can lead to the same number of added neutrons (*N*), for example, the KGS peptide YFHHNSDFYIPK (Figure 4). The default assumption for this peptide is that it contains a ^15^N-Ser and a ^15^N-Lys, but when the simulations were fit to the data, there was unaccounted for abundance in the third through sixth isotopes (Figure 4A), which could indicate excess labeling. Subedi et al. determined through NMR experiments^18^ that Ser and Gly can be interconverted by HEK293 cells, likely via serine hydroxymethyltransferase, so the [^15^N, ^13^C_2_]-Gly could be converted to a [^15^N, ^13^C_2_]-Ser and incorporate unintended ^13^C labels. Also, there is a possibility that Lys could contain two ^15^N, if the ε nitrogen does not undergo metabolic scrambling. Two possible routes can yield A + 3 contributions, either [^15^N_3_] or [^15^N, ^13^C_2_], which may be discriminated by their mass defects. The fitting process was performed two times, each with a different assumption for the *N* = 3 labeling state, and a single-point internal calibration was applied to the MS data at the monoisotopic peak to give a mass error of less than 0.1 ppm. After the intensity was fit, the mass errors between the third isotope peak in the simulation for [^15^N_3_] and [^15^N, ^13^C_2_] were −1.86 and 0.04 ppm, respectively. Similarly, the fourth isotope peak could arise from [^15^N_4_] or [^15^N_2_, ^13^C_2_], which give mass errors of −4.63 and 0.45 ppm, respectively. This gives good evidence that 15% of the Ser sampled in this spectrum are [^15^N, ^13^C_2_]-Ser, which were converted from the [^15^N, ^13^C_2_]-Gly used in the growth medium. This discrimination at this resolution (∼100,000) is only possible because the underlying contributions of ^15^N and ^13^C to the isotopic fine structure of each peak shift its centroid depending on the label that is present.

**Figure 5:**
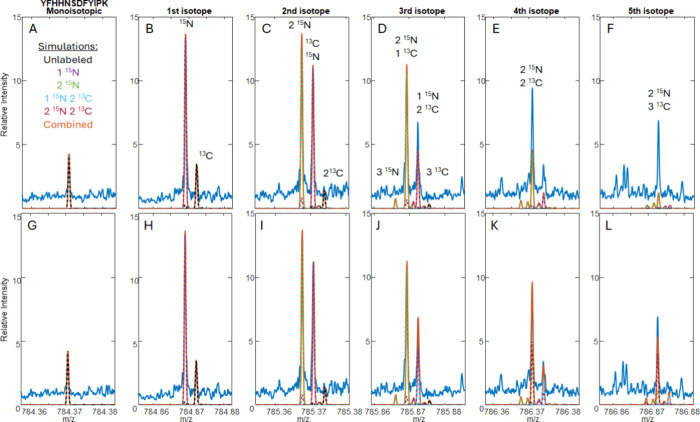
High-resolution mass spectra for YFHHNSDFYIPK (KGS) overlaid with simulated isotopic fine structure patterns (A–F) assuming a maximum of two ^15^N and (G–L) with the addition of simulations containing [^15^N, ^13^C_2_] (dotted light blue) and [^15^N_2_, ^13^C_2_] (dotted maroon). The combined isotope pattern simulation is shown in orange.

Measuring the isotopic fine structure directly can remove the ambiguity and guesswork, as the ^15^N and ^13^C contributions can be resolved from each other. Figure 5 shows the same peptide, YFHHNSDFYIPK, measured at 1.4 M resolution with simulations for the unlabeled peptide (yellow) and the peptide containing one ^15^N label (purple) and two ^15^N labels (green). Notice that there is hardly any overlap between these simulations, making it almost unnecessary to generate a linear combination. When these simulations are plotted over the data, they account for all of the observed abundance in the first and second isotope peak clusters (Figure 5B,C), which gives high confidence that ^15^N labels are the main contributors to the A + 1 and A + 2 isotopes. Next, there are two labeling options to arrive at A + 3, either ^15^N_3_ or [^15^N, ^13^C_2_]. Looking at the third isotopic cluster (Figure 5D), there is no peak corresponding to ^15^N_3,_ but there is a peak for [^15^N, ^13^C_2_], which has not been fully accounted for by the simulations. This confirms that [^15^N, ^13^C_2_]-Ser is the likely assignment for the extra labels. Estimates for the incorporation of each labeling state differed from the measurements of this peptide with unresolved fine structure. In both spectra, the unlabeled peptide contributed 14% of the total peptide population. The peptide containing one ^15^N contributed 30% of the isotope distribution but only 19% of the fine structure distribution. The peptide containing two ^15^N labels was found to be 21% of the population using the low-resolution spectrum but over double that (44%) from fitting using the fine structure. These inconsistencies are likely due to the low abundance (<5%) of the peptide in the fine-structure spectrum, which leads to nonstatistical sampling of ions. The abundance of fine-structure peaks in FT-ICR can also be distorted by apodization and the Fourier transform itself.^22^

### Localizing Labels with MS/MS

Aside from the percent incorporation of a label, it is also important to determine the location of the label and whether there are labels in off-target locations. Tandem mass spectrometry can be used for locating the positions of isotope labels. For these selectively labeled GFP samples, the quantity of protein available to us was limited, so we opted to acquire CID spectra using data-dependent acquisition (DDA) using CE-MS. Each CE-MS run consumed less than 200 nL of sample, which is desirable to sequence a large number of peptides with limited sample availability. Following the CE-MS run, several labeled peptides were identified using the in-house peptide mass fingerprinting software, and then their corresponding CID spectra were exported and analyzed using the MS/MS analysis software in Figure S1.

**Figure 6:**
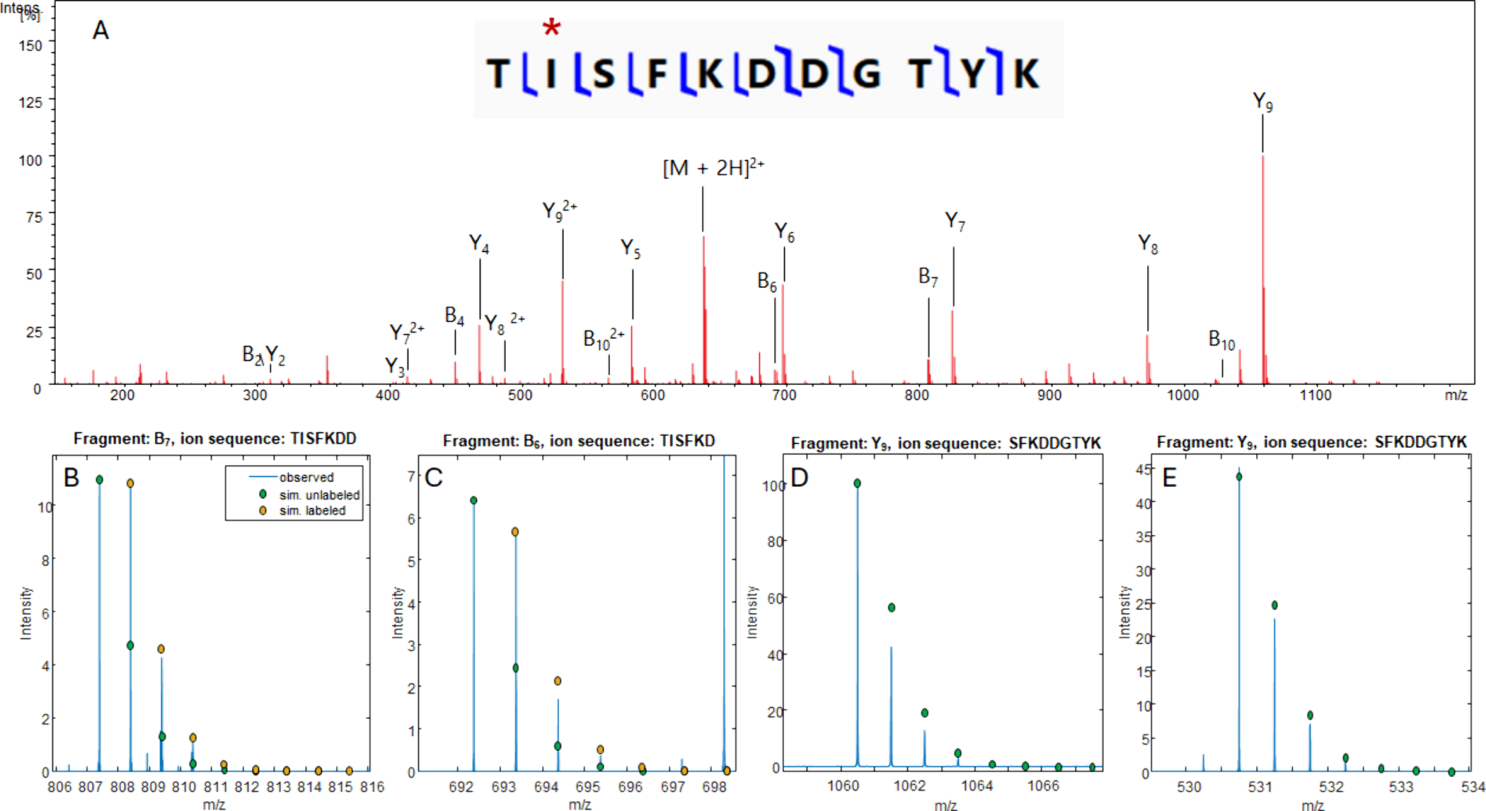
(A) CID spectrum for the peptide TISFKDDGTYK (VIL), which contains one ^15^N label with a fragmentation map generated in Prosight Lite. (B, C) Expansions of the B_7_ and B_6_ fragments, which contain a ^15^N-Ile, overlaid with simulated isotope patterns for the unlabeled fragment (green) and the labeled fragment (yellow). (D, E) Expansions of the Y_9_^2+^ and Y_9_^+^ ions, which do not contain an isotope label, overlaid with isotope pattern simulations for the unlabeled fragment (green).

Figure 6a shows a CID spectrum for the peptide TISFKDDGTYK that was extracted from the CE-MS dataset for the GFP-VIL construct. This peptide should contain a single ^15^N label in isoleucine. Table S5 shows fragment ion matches for all B and Y that were generated from our MS/MS software using a 5 ppm error tolerance. 90% sequence coverage was achieved using the unlabeled peptide peaks (Prosight Lite). All the B ions that were detected contained a ^15^N-Ile, while none of the Y ions were expected to contain a label. The B_4_ and B_10_ ions are likely false positives, as their unlabeled peaks are >800 ppm away from theoretical values, and the labeled peaks were >2 ppm away, while nearly all other fragments had mass errors of <1 ppm in this dataset. Visual inspection of the B ions (Figures 6b,c and S10) confirms the presence of a ^15^N label from their higher-than-expected A + 1 isotope abundance compared to simulation. None of the detected Y ions had an A + 1 peak intensity that exceeded the intensity of the A + 1 peak in the simulation, which indicated that there were no ^15^N labels in non-Ile residues. Interestingly, some of the A + 1 peaks fell below the expected intensity in the simulation (Figure 6d), even for high-intensity fragments. Only two MS^2^ scans were obtained for this peptide; therefore, the deviation in intensities could be due to low signal averaging and undersampling of the ion population. Also, some of the isotope peaks have partially resolved fine structures, which could also reduce the intensity of the observed peak.

**Figure 7:**
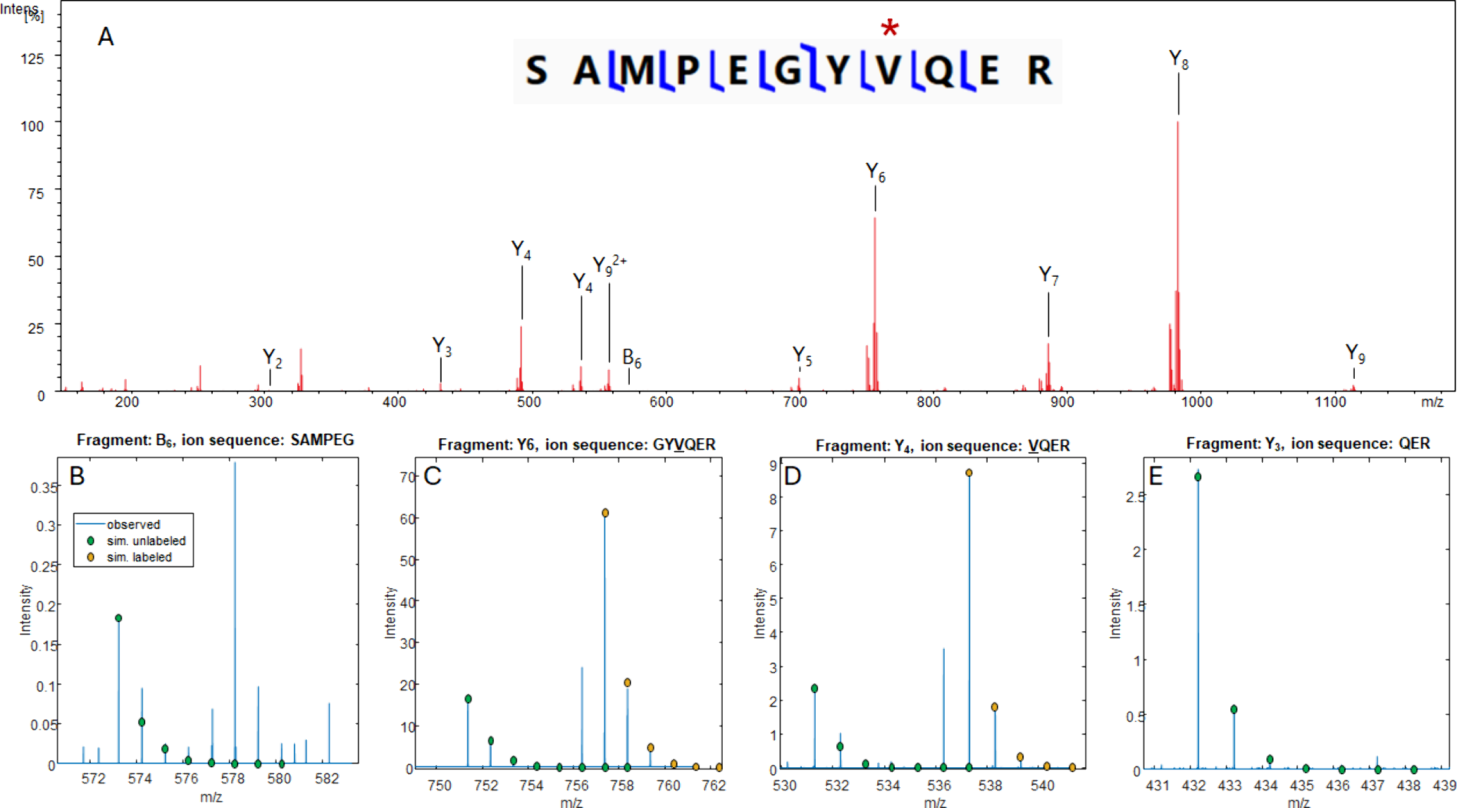
(A) CID spectrum of the peptide SAMPEGYVQER and a fragmentation map generated in Prosight Light, assuming the presence of one ^15^N label and five ^13^C labels. (B) Expansion of the B_6_ ion, which should not contain any labels yet displays higher than expected abundance of the A + 1 and A + 5 peaks. (C, D) Expansions of the Y_6_ and Y_4_ ions, which contain a labeled valine and have the same isotope pattern as the precursor ion, overlaid with isotope pattern simulations of the unlabeled fragment (green) and labeled fragment (yellow). (E) Expansion of the Y_3_ ion, which has been cleaved postvaline and has the expected isotope pattern of an unlabeled fragment (green).

A similar analysis was performed for SAMPEGYVQER, also from the VIL construct (Figure 7). This is the same peptide that was fit in Figure 2B and Figure 3, which has one ^15^N label and five ^13^C labels in valine. This peptide was of particular interest because it appears to have an intermediately labeled state with one ^15^N label removed (A + 5 from the monoisotopic peak) in addition to the fully labeled peak at A + 6. Alanine was also identified as a possible recipient of ^15^N labels by Subedi et al.^18^ CID of SAMPEGYVQER yielded primarily Y ions and a single B ion (Figure 7A and Table S6) with sequence coverage of 70%. The same isotope pattern that was seen in the precursor ion could also be seen in the Y fragments containing valine (Figure 7C,D). For Y fragments that do not contain valine, the distributions at A + 5 and A + 6 are absent (Figure 7E), confirming the locations of the ^15^N and ^13^C labels as well as the hypothesis that valine loses a ^15^N due to metabolic scrambling of its α-amino group. Only the B_6_ ion contained an alanine (Figure 7b), but it unexpectedly contained A + 5 and A + 6 peaks, suggesting that a labeled valine was present. The B_6_ ion intensity is very close to the baseline; therefore, these assignments are not completely conclusive.

In the future, it should be possible to fit simulations of the various labeling states to the fragment ions as well, although this feature has not yet been implemented in the software we have developed. This particular CZE-MS dataset would not be a good candidate to perform the fitting process, as each peptide only appears in two to four MS1 spectra and is only selected for MS/MS for one to two scans on average. As discussed in a previous article,^23^ signal averaging is an important consideration to obtain statistically relevant isotope distributions. As discussed above, large deviations were already observed between simulations for unlabeled peptides and their observed isotope patterns, so the fits obtained from this dataset would likely have large deviations.

## Conclusions

The incorporation of selective isotope labels in peptides can be quantitatively assigned by fitting linear combinations of many simulated isotope patterns to experimental MS data, and this is an especially useful strategy when the isotope patterns of the labeled and unlabeled patterns overlap. Peptides that contain only one type of label can be fit simply on most types of MS instrumentation. If both ^15^N and ^13^C are present, different label compositions can lead to the same number of added isotopes. Mass accuracy better than 1 ppm allows for incorrect label compositions to be discredited due to their isotopic mass defects. Isotopic fine structure measurements simplify this analysis, as the different labeling compositions can be resolved from each other, which greatly simplifies the fitting process. High signal-to-noise ratio and sufficient signal averaging are important to sample a large enough population of ions to produce a statistically relevant distribution of isotopes. Tandem mass spectra can also be used to assess the quality of the labeling. While we did not perform any fitting on MS/MS spectra because the online CZE-MS had low signal averaging for each spectrum, this could be a route for experimentation in the future. The level of accuracy of the assignments might seem excessive for the application to NMR, but it should be noted that if metabolic scrambling is happening, this high accuracy is important. Scrambling causes extra signals to appear in the NMR spectrum, but because the scrambled labels have levels of incorporation different from those of the intentional labels, they have different intensities. Knowing the enrichment value to an integer value for each amino acid helps to assign signals in the NMR spectra because their intensities are proportional to the label incorporation.

## Data Availability

The modified version of MATLAB’s isotope simulator, isotopicdist, is available at https://github.com/erobertsFTMS/Sparse-isotope-labeling. The MATLAB software for determining the percent incorporation of sparse isotope labels in peptides is available at https://github.com/erobertsFTMS/Sparse-isotope-labeling. The software for analyzing MS/MS spectra of sparse isotope labeled peptides is available at https://github.com/erobertsFTMS/Sparse-isotope-labeling.

## Supporting Information Available

Software workflow for analyzing MS/MS spectra of sparse isotope labeled peptides, software workflow for fitting multiple isotopically labeled isotope patterns to experimental data, MALDI mass spectrum of GFP-CD16 (VIL) tryptic digest, table of identified GFP-CD16 (VIL) peptides, expansions of the isotope patterns of each GFP-CD16 (VIL) peptide, MALDI MS1 spectrum of a GFP-CD-16 (KGS) tryptic digest, table of identified GFP-CD16 (KGS) peptides, expansions of the isotope patterns of each GFP-CD16 (KGS) peptide, ESI mass spectrum of GFP-CD16 (VIL) tryptic digest, table of identified peptides from GFP-CD16 (VIL) ESI spectrum, expansions of the isotope patterns for each peptide in the GFP-CD16 (VIL) ESI spectrum, fitting results for GEELFTGVVPILVELDGDVNGHK (VIL), table with fitting results for GEELFTGVVPILVELDGDVNGHK (VIL), table with fragment ion assignments for TISFKDDGTYK (VIL), expansions of the isotope patterns for the fragment ions of TISFKDDGTYK (VIL), table with fragment ion assignments for SAMPEGYVQER (VIL), expansions of the isotope patterns for the fragment ions of SAMPEGYVQER (VIL), table of identified peptides for a GFP-CD16 (VIL) digest acquired using CZE-MS and processed with absorption mode using the SpectroSwiss FTMS booster, and expansions of the GFP-CD16 (VIL) peptides from the CZE-MS run (PDF)
