# Supplementary material for: Measuring 15N and 13C Enrichment Levels in Sparsely Labeled Proteins Using High-Resolution and Tandem Mass Spectrometry
Source: J Am Soc Mass Spectrom. 2024 Nov 12;35(12):2877–89. doi: 10.1021/jasms.4c00237 (PMC11622383; doi:10.1021/jasms.4c00237)
Supplement: Supplementary file 1 — js4c00237_si_001.pdf [file js4c00237_si_001.pdf]

SUPPORTING INFORMATION

Measuring ^{15}N and ^{13}C Enrichment Levels in Sparsely Labeled Proteins Using High-Resolution and Tandem Mass Spectrometry

Elijah T. Roberts,[1] Jonathan Choi,[1] Jeremy Risher,[1] Paul G Kremer,[2] Adam W. Barb,[1,2,3] I. Jonathan Amster[1*]

[1] Department of Chemistry, University of Georgia, Athens, GA 30602, USA

[2] Department of Biochemistry and Molecular Biology, University of Georgia, Athens, GA 30602, USA

[3] Complex Carbohydrate Research Center, University of Georgia, Athens, GA 30602, USA

Running Title: Selective Isotope Labeling Measurement and Simulation

<u>*Contact information for corresponding author:</u>

Department of Chemistry

1040K iSTEM-1

302 East Campus Road

University of Georgia

Athens, GA 30602-1546

Phone: 706-542-2001

Email: jamster@uga.edu

CONTENTS

PAGE

Figure S1: Software workflow for analyzing labeled peptide fragments ………………………………. S-3

Figure S2: Software workflow for fitting multiple isotope pattern simulations to data …..…………… S-4

Figure S3: MALDI MS1 spectrum of GFP-CD16 (VIL) peptides ………………………...………… S-5

Figure S4: Expansions of GFP-CD16 (VIL) peptide isotope patterns ………………………….……… S-6

Figure S5: MALDI MS1 spectrum of GFP-CD16 (KGS) ………………………………..……….. S-7

Figure S6: Expansions of GFP-CD16 (KGS) peptide isotope patterns ……………………...………. S-8

Figure S7: ESI spectrum of GFP-CD16 (VIL) peptides ……………………………………….……. S-9

Figure S8: Expansions of GFP-CD16 (VIL) peptide isotope patterns from figure S7 ………….…… S-10

Figure S9: Fitting results for GEELFTGVVPILVELDGDVNGHK …………………..…………… S-11

Figure S10: Expansions of TISFLDDGTYK (VIL) fragment ion isotope patterns …………..………. S-12

Figure S11: Expansions of SAMPEGYVQER (VIL) fragment ion isotope patterns ….……..……….. S-13

Figure S12: Expansions of GFP-CD16 (VIL) peptides using CZE-MS ………………..………….. S-14

Table S1: MS1 results for GFP-CD16 (VIL) peptides using MALDI …………………..…………… S-15

Table S2: MS1 results for GFP-CD16 (KGS) peptides using MALDI ……………………………….. S-16

Table S3: MS1 results for GFP-CD16 (VIL) peptides using ESI ……………………..…………..…… S-16

Table S4: Fitting results for GEELFTGVVPILVELDGDVNGHK …………………….………….. S-17

Table S5: Fragment ion assignments for TISFKDDGTYK (VIL) ……………………………………. S-18

Table S6: Fragment ion assignments for SAMPEGYVQER (VIL) ……………………..…………. S-18

Table S7: MS1 results for GFP-CD16 (VIL) peptides using CZE-MS …………………….………. S-19

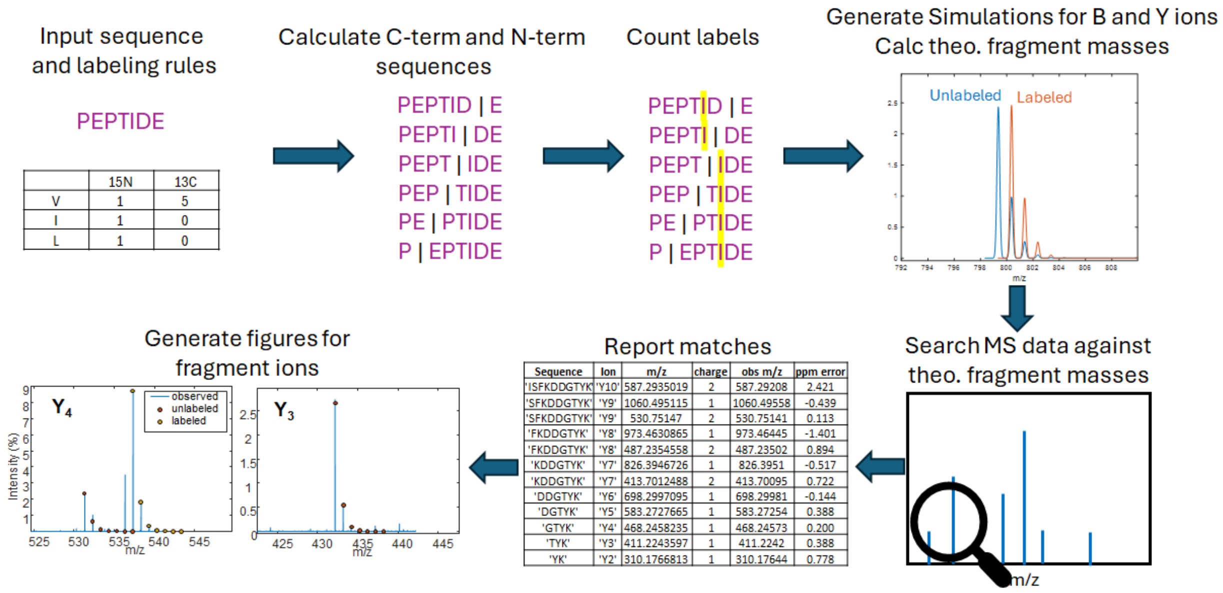

Figure S1: Software workflow for analyzing CID spectra of isotopically labeled peptides.

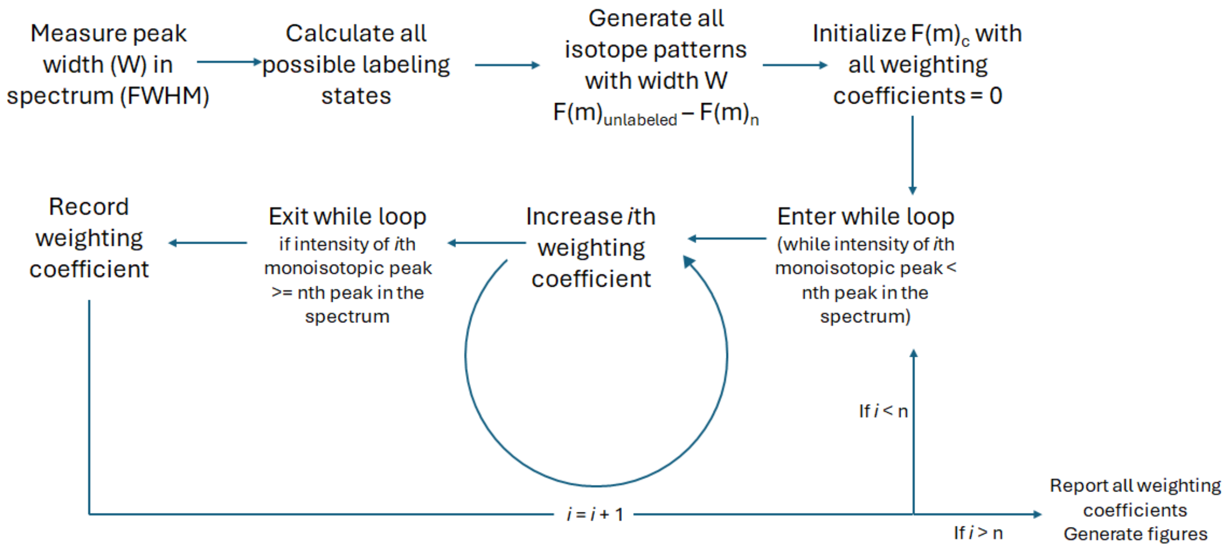

Figure S2: Software workflow for fitting multiple isotope pattern simulations to experimental spectra of selectively isotope labeled peptides.

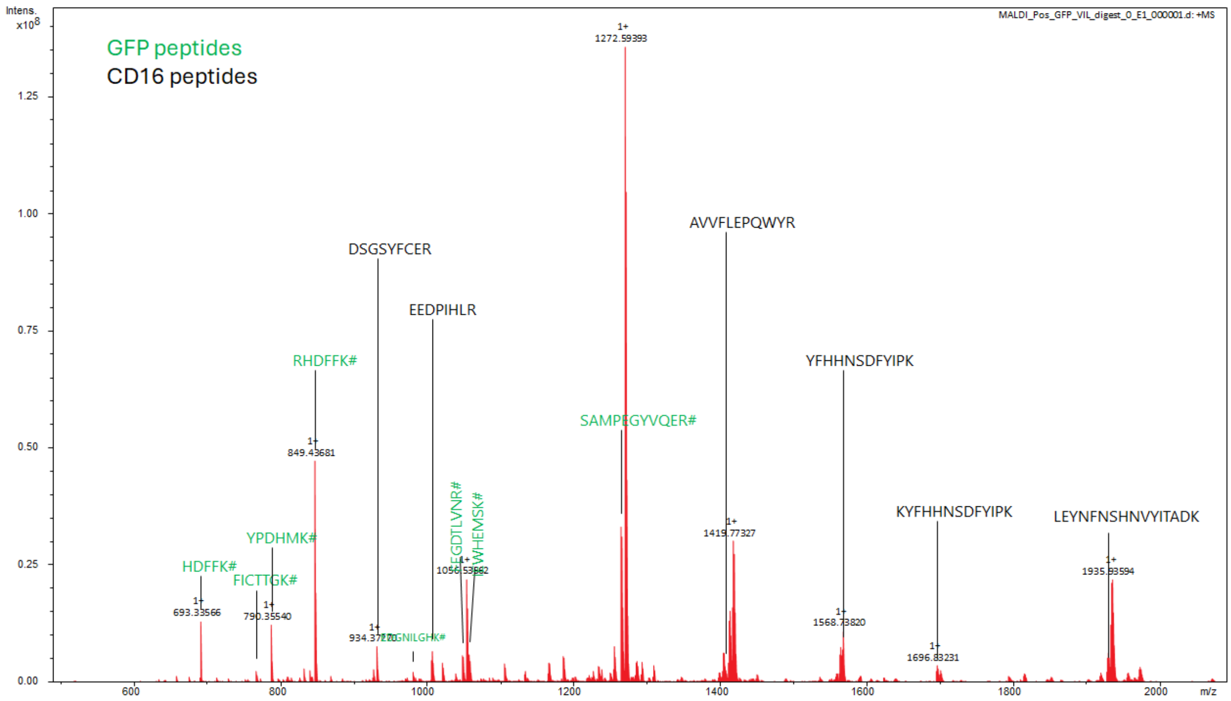

Figure S3: MALDI MS1 spectrum of a GFP-CD16 (VIL) tryptic digest.

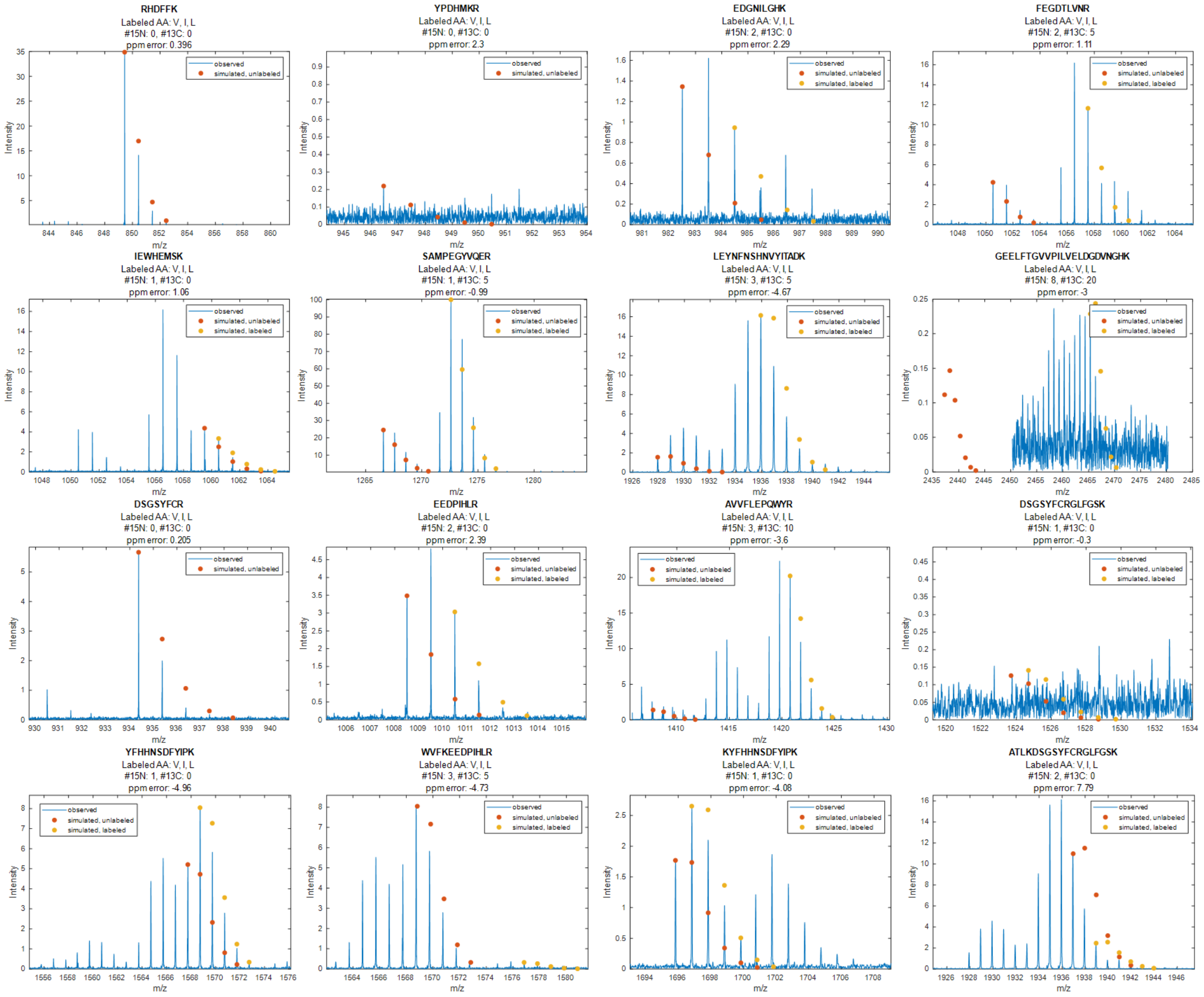

Figure S4: Expansions of the GFP-CD16 (VIL) tryptic peptides from Figure S4 and Table S1. Each Isotope pattern is overlaid with an simulation for an unlabeled (orange) peptide and a fully labeled (yellow) assuming the number of labels shown in Table S1.

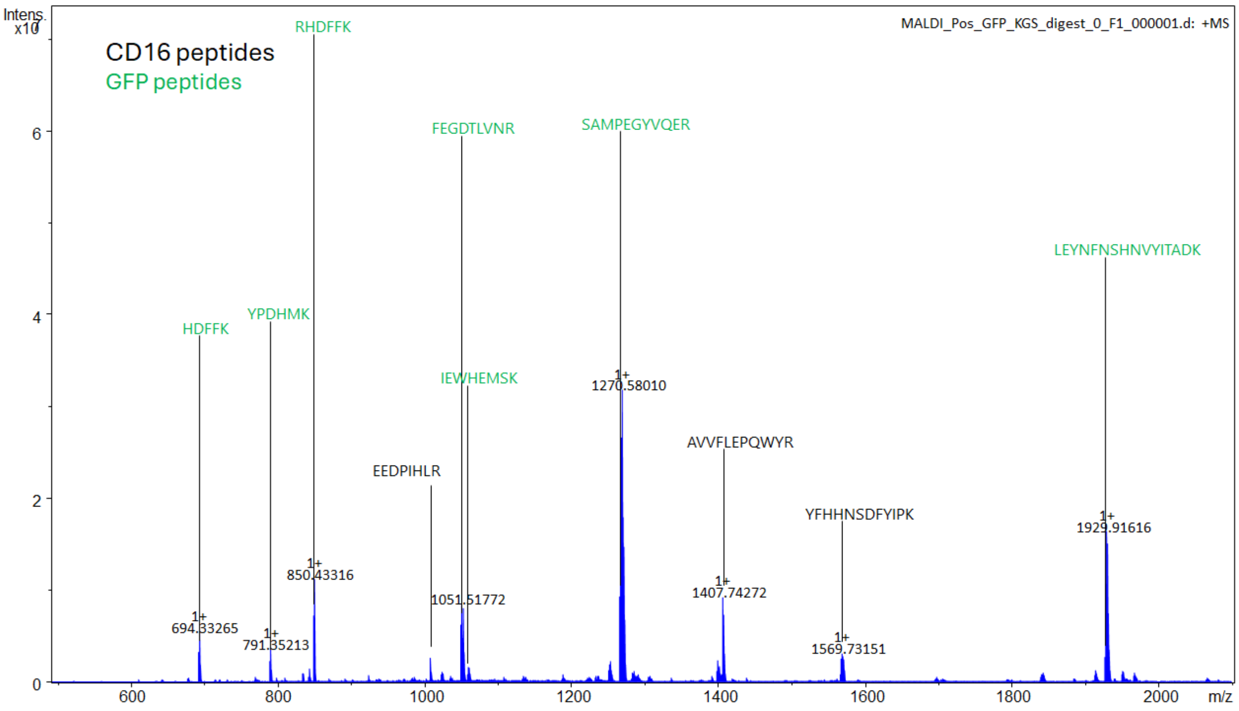

Figure S5: MALDI MS1 spectrum of a GFP-CD16 (KGS) tryptic digest.

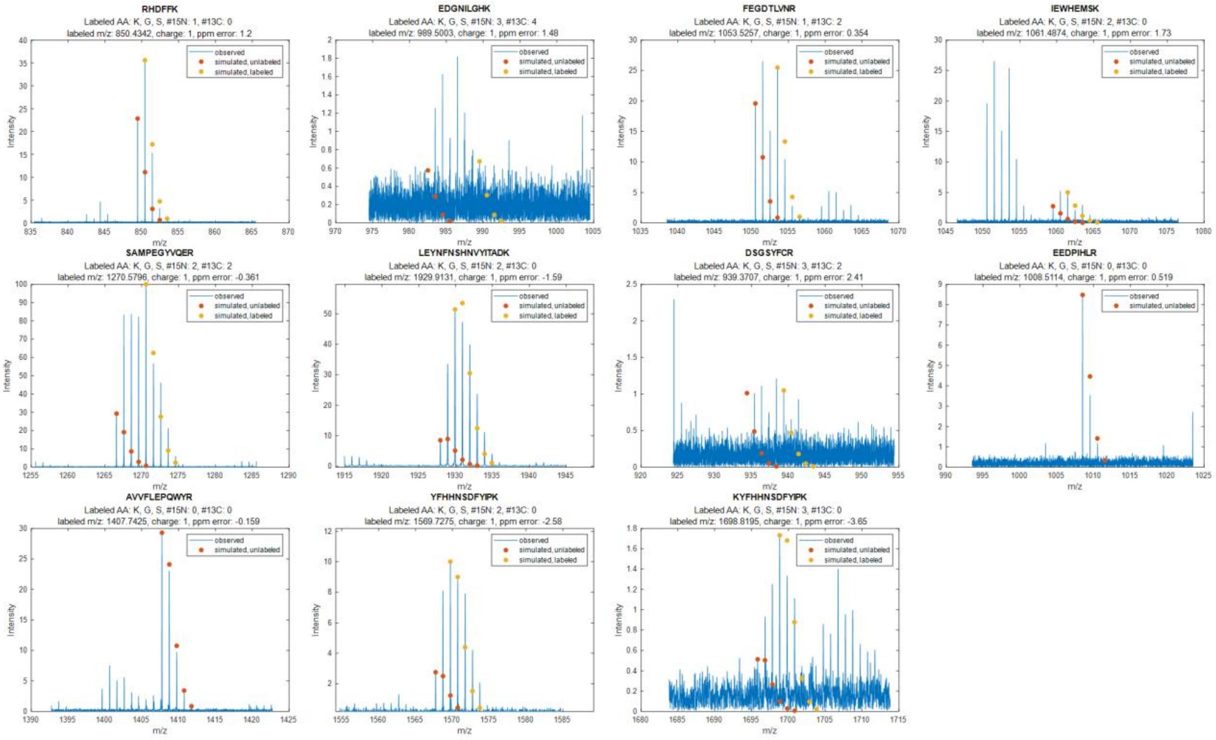

Figure S6: Expansions of the GFP-CD16 (VIL) peptides identified from Figure S5 and Table S3. Each Isotope pattern is overlaid with a simulation for an unlabeled (orange) peptide and a fully labeled (yellow) assuming the number of labels shown in Table S3.

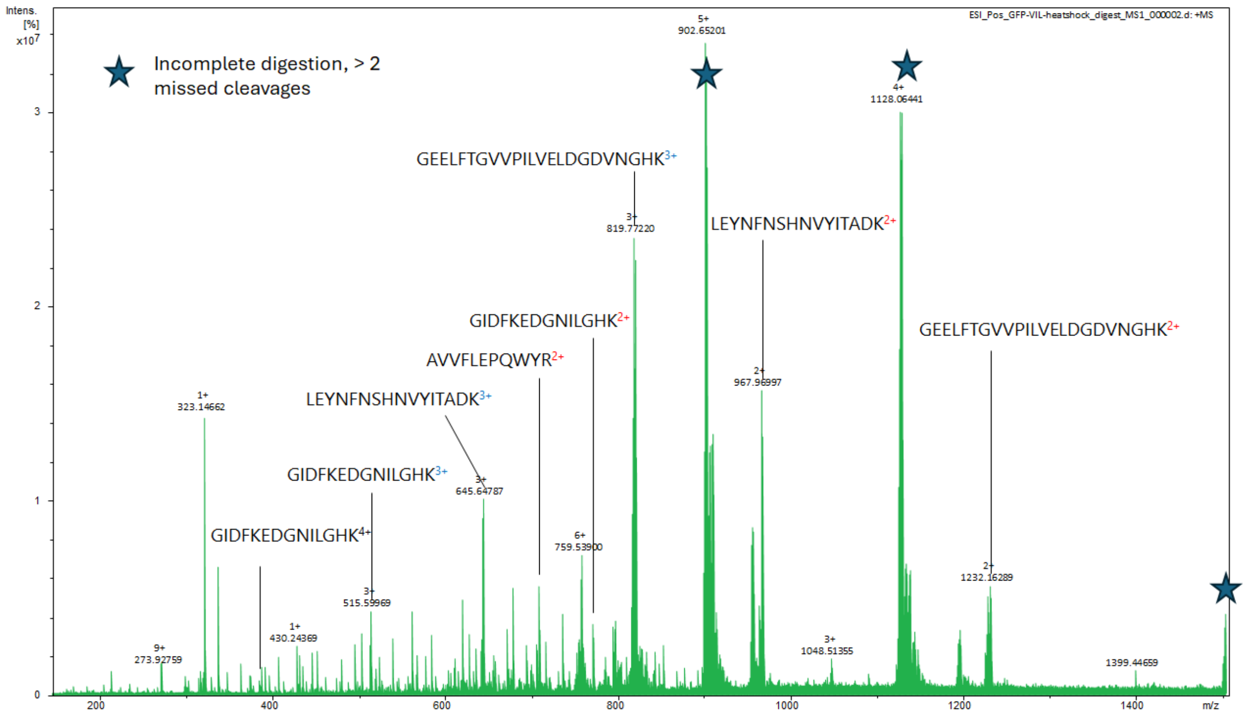

Figure S7: ESI mass spectrum of a GFP-CD16 (VIL) tryptic digest.

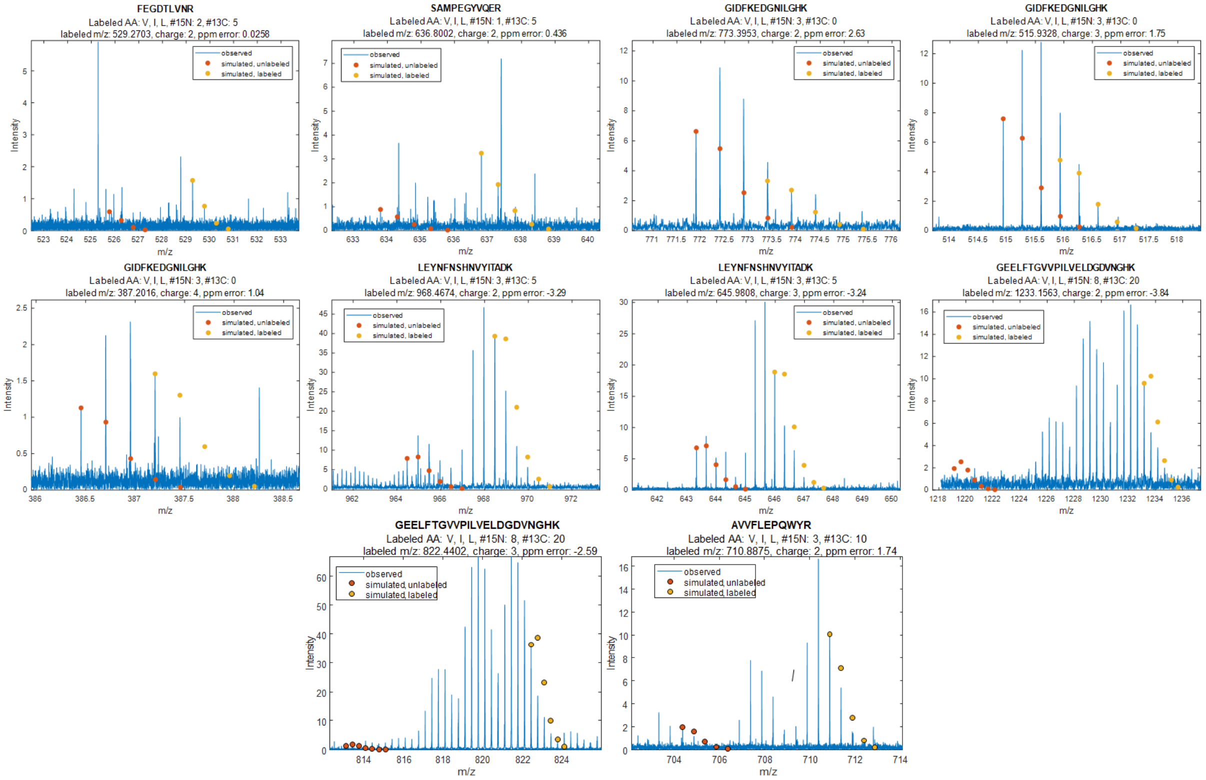

Figure S8: Expansions of the GFP-CD16 (VIL) tryptic peptides from Figure S7 and Table S3. Each Isotope pattern is overlaid with an simulation for an unlabeled (orange) peptide and a fully labeled (yellow) assuming the number of labels shown in Table S3.

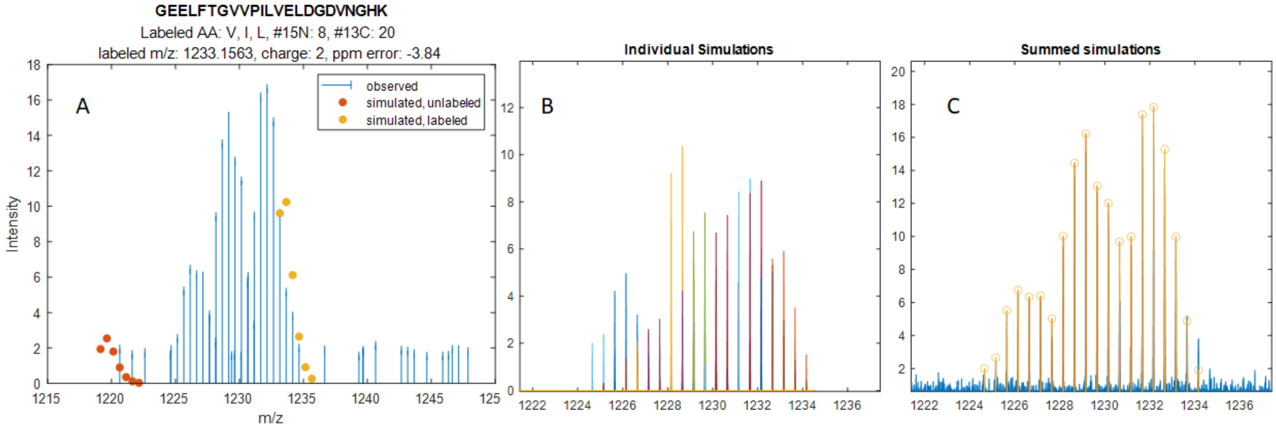

Figure S9: A) Mass spectrum (blue) of a GFP-CD16 peptide which should contain 8 ^{15}N and 20 ^{13}C. Isotope patterns for the unlabeled and fully labeled peptide are shown in orange and yellow, respectively. B) Overlay of many intermediately labeled isotope pattern simulations. C) Simulated isotope pattern generated from a linear combination of the simulations in B (orange) overlaid with the experimental data (blue).

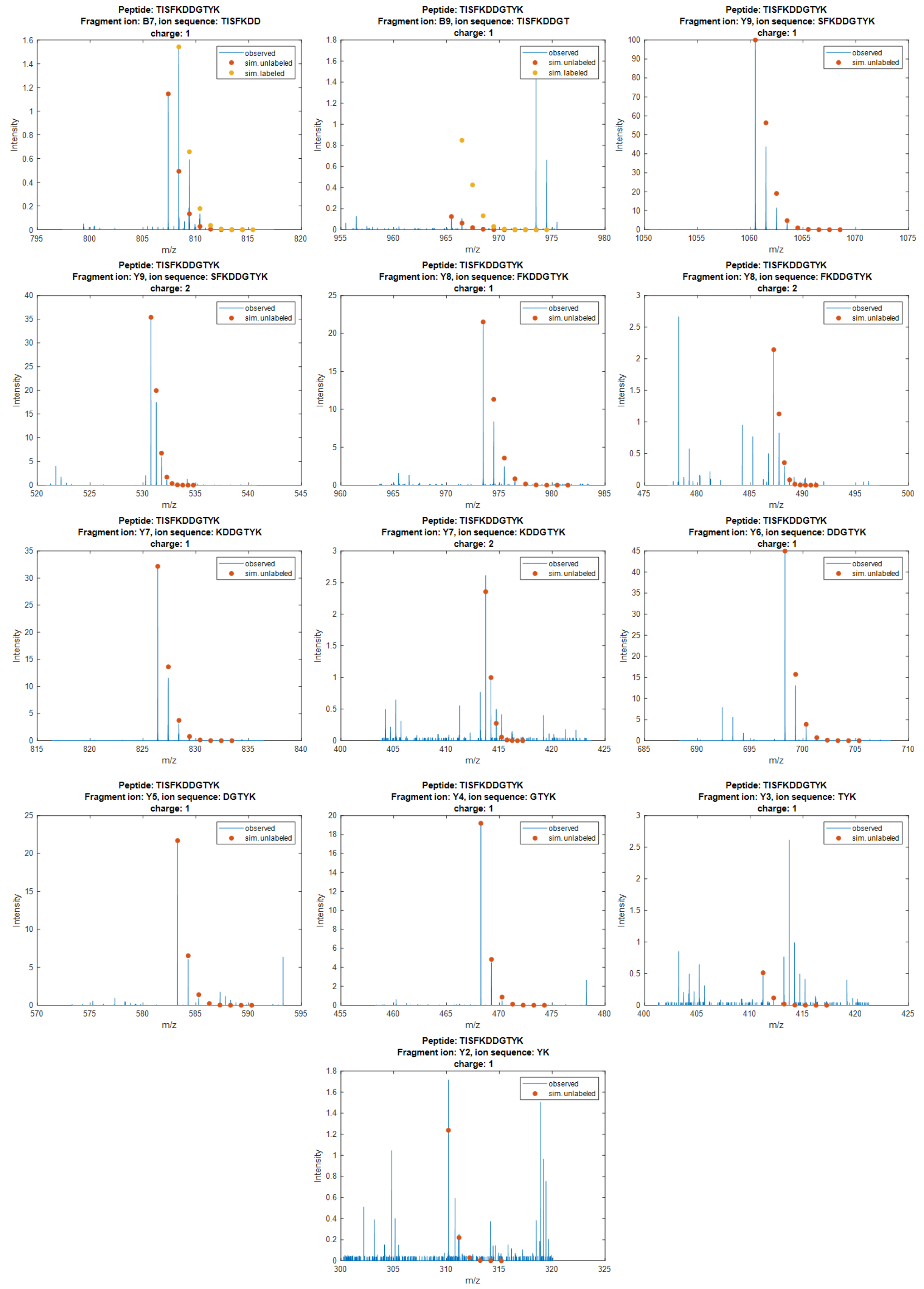

Figure S10: Expansions of the TISFLDDGTYK (VIL) fragment ions identified in Table S4. Each isotope pattern is overlaid with a simulation for an unlabeled fragment (orange) and if applicable, a labeled fragment (yellow).

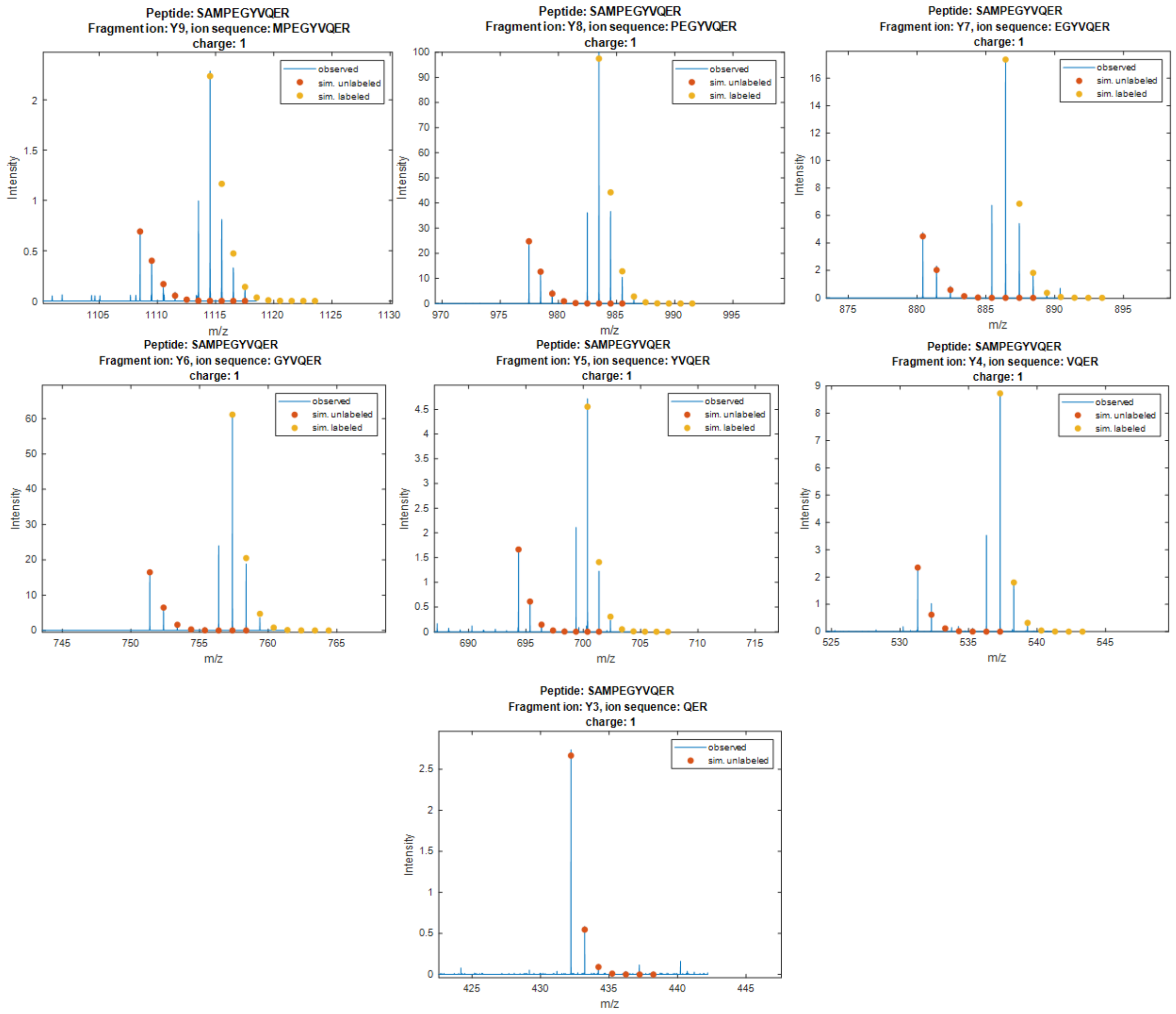

Figure S11: Expansions of the SAMPEGYVQER (VIL) fragment ions identified in Table S5. Each isotope pattern is overlaid with a simulation for an unlabeled fragment (orange) and if applicable, a labeled fragment (yellow)

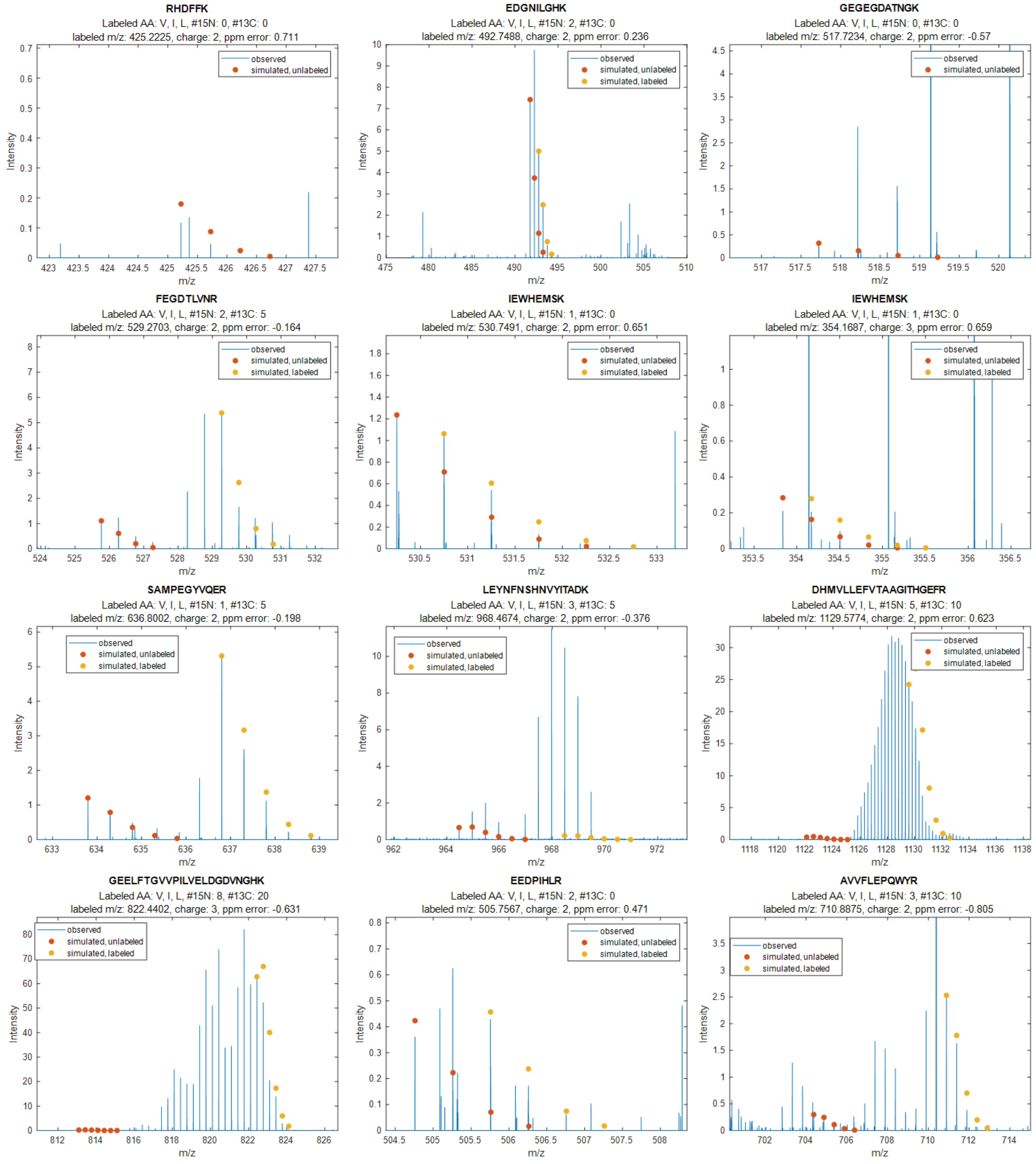

Figure S12: Expansions of the GFP-CD16 (VIL) peptides that were identified in Table S5.

Table S1: Sparse labeled GFP-CD16 (VIL) peptide matches at the MS1 level.

Peptide	Unlabeled m/z	charge	V	I	L	#15N	#13C	Theo Labeled m/z	obs m/z	ppm error
RHDFFK	849.4371465	1	0	0	0	0	0	849.4371465	849.43681	0.396
YPDHMKR	946.4568957	1	0	0	0	0	0	946.4568957	946.45472	2.299
EDGNILGHK	982.4957836	1	0	1	1	2	0	984.4898534	984.4876	2.289
FEGDTLVNR	1050.521998	1	1	0	1	2	5	1057.532842	1057.53167	1.109
IEWHEMSK	1059.493341	1	0	1	0	1	0	1060.490376	1060.48925	1.061
SAMPEGYVQER	1266.578861	1	1	0	0	1	5	1272.59267	1272.59393	-0.990
LEYNFNSHNVYITADK	1927.919017	1	1	1	1	3	5	1935.926895	1935.93594	-4.672
GEELFTGVVPILVELDGDVNGHK	2437.26148	1	4	1	3	8	20	2465.304856	2465.31226	-3.003
DSGSYFCR	934.3728913	1	0	0	0	0	0	934.3728913	934.3727	0.205
EEDPIHLR	1008.511434	1	0	1	1	2	0	1010.505503	1010.50309	2.388
AVVFLEPQWYR	1407.742496	1	2	0	1	3	10	1420.767149	1420.77227	-3.604
DSGSYFCRGLFGSK	1523.695288	1	0	0	1	1	0	1524.692323	1524.69278	-0.300
YFHHNSDFYIPK	1567.733388	1	0	1	0	1	0	1568.730423	1568.7382	-4.958
WVFKEEDPIHLR	1568.822537	1	1	1	1	3	5	1576.830416	1576.83787	-4.727
KYFHHNSDFYIPK	1695.828351	1	0	1	0	1	0	1696.825386	1696.83231	-4.081
ATLKDSGSYFCRGLFGSK	1936.959107	1	0	0	2	2	0	1938.953177	1938.93808	7.786

Table S2: Sparse labeled GFP-CD16 (KGS) peptide matches at the MS1 level.

Sequence	Theo. Unlabeled m/z	Charge	K	G	S	15N	13C	Theo labeled m/z	obs m/z	ppm error
'RHDFFK'	849.4371465	1	1	0	0	1	0	850.4341814	850.43316	1.20
'EDGNILGHK'	982.4957836	1	1	2	0	3	4	989.5003076	989.49884	1.48
'FEGDTLVNR'	1050.521998	1	0	1	0	1	2	1053.525743	1053.52537	0.35
'IEWHEMSK'	1059.493341	1	1	0	1	2	0	1061.487411	1061.48557	1.73
'SAMPEGYVQER'	1266.578861	1	0	1	1	2	2	1270.579641	1270.5801	-0.36
'LEYNFNSHNVYITADK'	1927.919017	1	1	0	1	2	0	1929.913086	1929.91616	-1.59
'DSGSYFCR'	934.3728913	1	0	1	2	3	2	939.3707056	939.36844	2.41
'EEDPIHLR'	1008.511434	1	0	0	0	0	0	1008.511434	1008.51091	0.52
'AVVFLEPQWYR'	1407.742496	1	0	0	0	0	0	1407.742496	1407.74272	-0.16
'YFHHNSDFYIPK'	1567.733388	1	1	0	1	2	0	1569.727458	1569.73151	-2.58
'KYFHHNSDFYIPK'	1695.828351	1	2	0	1	3	0	1698.819456	1698.82565	-3.65

Table S3: *Sparse labeled GFP-CD16 (VIL) peptide matches at the MS1 level from the ESI spectrum in Figure S5.*

Peptide	Unlabeled m/z	charge	V	I	L	#15N	#13C	Theo Labeled m/z	obs m/z	ppm error
SAMPEGYVQER	633.7933432	2	1	0	0	1	5	636.8002477	636.79997	0.436
TISFKDDGTYK	637.8173412	2	0	1	0	1	0	638.3158586	638.31905	-5.000
GIDFKEDGNILGHK	771.8997281	2	0	2	1	3	0	773.3952805	773.39325	2.625
GIDFKEDGNILGHK	514.9357604	3	0	2	1	3	0	515.9327953	515.93189	1.755
GIDFKEDGNILGHK	386.4537766	4	0	2	1	3	0	387.2015528	387.20115	1.040
LEYNFNSHNVYITADK	964.4634208	2	1	1	1	3	5	968.4673603	968.47055	-3.294
LEYNFNSHNVYITADK	643.3115556	3	1	1	1	3	5	645.9808485	645.98294	-3.238
GEELFTGVVPILVELDGDVNGHK	1219.134653	2	4	1	3	8	20	1233.156341	1233.16107	-3.835
GEELFTGVVPILVELDGDVNGHK	813.0923767	3	4	1	3	8	20	822.4401687	822.4423	-2.591
GEELFTGVVPILVELDGDVNGHKFSVR	488.5950954	6	5	1	3	9	25	494.2712929	494.26993	2.757
GMRTEDLPK	523.7691398	2	0	0	1	1	0	524.2676572	524.27024	-4.926
AVVFLEPQWYR	704.3751606	2	2	0	1	3	10	710.8874872	710.88625	1.740

Table S4: All possible intermediate labeling combinations for the peptide GEELFTGVVPILVELDGDVNGHK from the VIL construct, and corresponding abundances for each labeling state as determined in the fitting process in Figure S8.

# 13C	# 15N	rel ab (Σ = 1)
0	0	0
1	0	0
2	0	0
3	0	0
4	0	0
5	0	0
6	0	0
7	0	0
8	0	0
0	5	0
1	5	0
2	5	0
3	5	0
4	5	0
5	5	0
6	5	0
7	5	0
8	5	0

# 13C	# 15N	rel ab (Σ = 1)
0	10	0
1	10	0.0318
2	10	0.0051
3	10	0.0663
4	10	0.0215
5	10	0.0327
6	10	0.0404
7	10	0
8	10	0
0	15	0
1	15	0
2	15	0
3	15	0.1382
4	15	0.0632
5	15	0.1006
6	15	0.0168
7	15	0.0992
8	15	0

# 13C	# 15N	rel ab (Σ = 1)
0	20	0
1	20	0
2	20	0
3	20	0
4	20	0.1199
5	20	0.1187
6	20	0.0667
7	20	0.0789
8	20	0.00

Table S5: Fragment Ion assignments for TISFKDDGTYK (VIL) obtained from a DDA CZE-MS experiment.

Ion sequence	Ion	Unlabeled m/z	Charge	obs m/z	ppm error	V	I	L	15N	13C	Theo. Labeled m/z	obs. m/z	ppm error
'SFKDDGTYK'	'Y9'	1060.495115	1	1060.49558	-0.438587687	0	0	0	0	0	1060.495115	1060.49558	-0.44
'SFKDDGTYK'	'Y9'	530.75147	2	530.75141	0.11296436	0	0	0	0	0	530.75147	530.75141	0.11
'FKDDGTYK'	'Y8'	973.4630865	1	973.46445	-1.400700262	0	0	0	0	0	973.4630865	973.46445	-1.40
'FKDDGTYK'	'Y8'	487.2354558	2	487.23502	0.894333622	0	0	0	0	0	487.2354558	487.23502	0.89
'KDDGTYK'	'Y7'	826.3946726	1	826.3951	-0.517242202	0	0	0	0	0	826.3946726	826.3951	-0.52
'KDDGTYK'	'Y7'	413.7012488	2	413.70095	0.722243288	0	0	0	0	0	413.7012488	413.70095	0.72
'DDGTYK'	'Y6'	698.2997095	1	698.29981	-0.143869314	0	0	0	0	0	698.2997095	698.29981	-0.14
'DGTYK'	'Y5'	583.2727665	1	583.27254	0.388333063	0	0	0	0	0	583.2727665	583.27254	0.39
'GTYK'	'Y4'	468.2458235	1	468.24573	0.199621855	0	0	0	0	0	468.2458235	468.24573	0.20
'TYK'	'Y3'	411.2243597	1	411.2242	0.388470421	0	0	0	0	0	411.2243597	411.2242	0.39
'YK'	'Y2'	310.1766813	1	310.17644	0.777861182	0	0	0	0	0	310.1766813	310.17644	0.78
'TISFKDD'	'B7'	807.3888589	1	808.38515	-1233.966873	0	1	0	1	0	808.3858938	808.38515	0.92
'TISFKDDGT'	'B9'	965.4580011	1	965.45625	1.813742388	0	1	0	1	0	966.455036	965.45625	1033.45

Table S6: Fragment Ion assignments for SAMPEGYVQER (VIL).

Ion sequence	Ion	Unlabeled m/z	Charge	obs m/z	ppm error	V	I	L	15N	13C	Theo. Labeled m/z	obs. m/z	ppm error
'MPEGYVQER'	'Y9'	1108.509719	1	1108.50821	1.36136524	1	0	0	1	5	1114.523528	1114.52275	0.6982
'MPEGYVQER'	'Y9'	554.7587721	2	554.7577	1.932478411	1	0	0	1	5	557.7656766	557.7655	0.3166
'PEGYVQER'	'Y8'	977.4692345	1	977.47027	-1.059388432	1	0	0	1	5	983.4830436	983.48331	-0.2709
'PEGYVQER'	'Y8'	489.2385298	2	489.23864	-0.225337424	1	0	0	1	5	492.2454343	492.24554	-0.2147
'EGYVQER'	'Y7'	880.4164706	1	880.41747	-1.13511234	1	0	0	1	5	886.4302797	886.43158	-1.4669
'GYVQER'	'Y6'	751.3738775	1	751.37202	2.472180968	1	0	0	1	5	757.3876866	757.3881	-0.5458
'YVQER'	'Y5'	694.3524138	1	694.35086	2.237780944	1	0	0	1	5	700.3662229	700.36615	0.1041
'VQER'	'Y4'	531.2890853	1	531.28902	0.122852703	1	0	0	1	5	537.3028944	537.30279	0.1942
'QER'	'Y3'	432.2206714	1	432.22052	0.350177837	0	0	0	0	0	432.2206714	432.22052	0.3502
'ER'	'Y2'	304.1620938	1	304.16185	0.801686683	0	0	0	0	0	304.1620938	304.16185	0.8017
'SAMPEG'	'B6'	573.2342725	1	573.23447	-0.344522666	0	0	0	0	0	573.2342725	573.23447	-0.344523

Table S7: MS1 results for the GFP-CD16 (VIL) digest acquired using CZE-MS with parallel absorption mode processing using the SpectroSwiss FTMS booster

Sequence	Theo. Unlabeled m/z	Charge	V	I	L	15N	13C	Theo labeled m/z	obs m/z	ppm error
'RHDFFK'	425.2224858	2	0	0	0	0	0	425.2224858	425.2221836	0.71
'EDGNILGHK'	491.7518043	2	0	1	1	2	0	492.7488392	492.748723	0.24
'GEGEGDATNGK'	517.7234408	2	0	0	0	0	0	517.7234408	517.7237357	-0.57
'FEGDTLVNR'	525.7649117	2	1	0	1	2	5	529.2703337	529.2704206	-0.16
'IEWHEMSK'	530.2505829	2	0	1	0	1	0	530.7491003	530.7487549	0.65
'IEWHEMSK'	353.8363303	3	0	1	0	1	0	354.1686752	354.1684418	0.66
'SAMPEGYVQER'	633.7933432	2	1	0	0	1	5	636.8002477	636.8003736	-0.20
'LEYNFNSHNVYITADK'	964.4634208	2	1	1	1	3	5	968.4673603	968.4677246	-0.38
'DHMVLLEFVTAAGITHGEFR'	1122.068061	2	2	1	2	5	10	1129.577423	1129.576719	0.62
'GEELFTGVVPILVELDGDVNGHK'	813.0923767	3	4	1	3	8	20	822.4401687	822.440688	-0.63
'EEDPIHLR'	504.7596293	2	0	1	1	2	0	505.7566642	505.7564258	0.47
'AVVFLEPQWYR'	704.3751606	2	2	0	1	3	10	710.8874872	710.8880593	-0.80
